# Semiquinone radical-bridged M_2_ (M = Fe, Co, Ni) complexes with strong magnetic exchange giving rise to slow magnetic relaxation†

**DOI:** 10.1039/d0sc03078c

**Published:** 2020-07-21

**Authors:** Khetpakorn Chakarawet, T. David Harris, Jeffrey R. Long

**Affiliations:** Department of Chemistry, University of California Berkeley Berkeley California 94720 USA jrlong@berkeley.edu; Department of Chemical and Biomolecular Engineering, University of California Berkeley Berkeley California 94720 USA; Materials Sciences Division, Lawrence Berkeley National Laboratory Berkeley California 94720 USA

## Abstract

The use of radical bridging ligands to facilitate strong magnetic exchange between paramagnetic metal centers represents a key step toward the realization of single-molecule magnets with high operating temperatures. Moreover, bridging ligands that allow the incorporation of high-anisotropy metal ions are particularly advantageous. Toward these ends, we report the synthesis and detailed characterization of the dinuclear hydroquinone-bridged complexes [(Me_6_tren)_2_M^II^_2_(C_6_H_4_O_2_^2−^)]^2+^ (Me_6_tren = tris(2-dimethylaminoethyl)amine; M = Fe, Co, Ni) and their one-electron-oxidized, semiquinone-bridged analogues [(Me_6_tren)_2_M^II^_2_(C_6_H_4_O_2_^−^˙)]^3+^. Single-crystal X-ray diffraction shows that the Me_6_tren ligand restrains the metal centers in a trigonal bipyramidal geometry, and coordination of the bridging hydro- or semiquinone ligand results in a parallel alignment of the three-fold axes. We quantify the *p*-benzosemiquinone–transition metal magnetic exchange coupling for the first time and find that the nickel(ii) complex exhibits a substantial *J* < −600 cm^−1^, resulting in a well-isolated *S* = 3/2 ground state even as high as 300 K. The iron and cobalt complexes feature metal–semiquinone exchange constants of *J* = −144(1) and −252(2) cm^−1^, respectively, which are substantially larger in magnitude than those reported for related bis(bidentate) semiquinoid complexes. Finally, the semiquinone-bridged cobalt and nickel complexes exhibit field-induced slow magnetic relaxation, with relaxation barriers of *U*_eff_ = 22 and 46 cm^−1^, respectively. Remarkably, the Orbach relaxation observed for the Ni complex is in stark contrast to the fast processes that dominate relaxation in related mononuclear Ni^II^ complexes, thus demonstrating that strong magnetic coupling can engender slow magnetic relaxation.

## Abstract 2

A semiquinone radical bridging two trigonal bipyramidal metal centers facilitates strong magnetic exchange and single-molecule magnet behavior.

## Graphical abstract

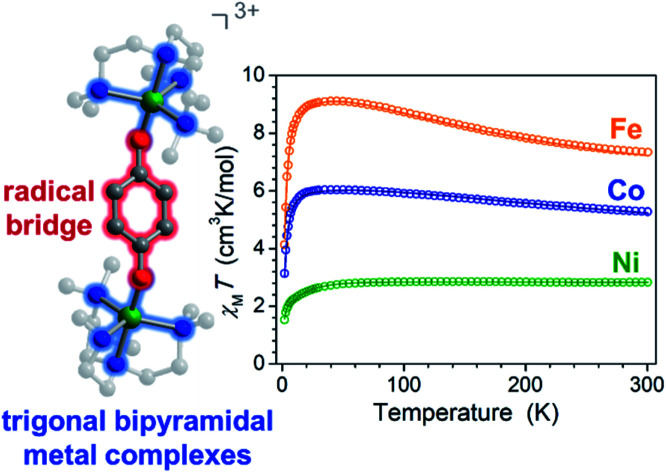

## Introduction

**Scheme 1:**
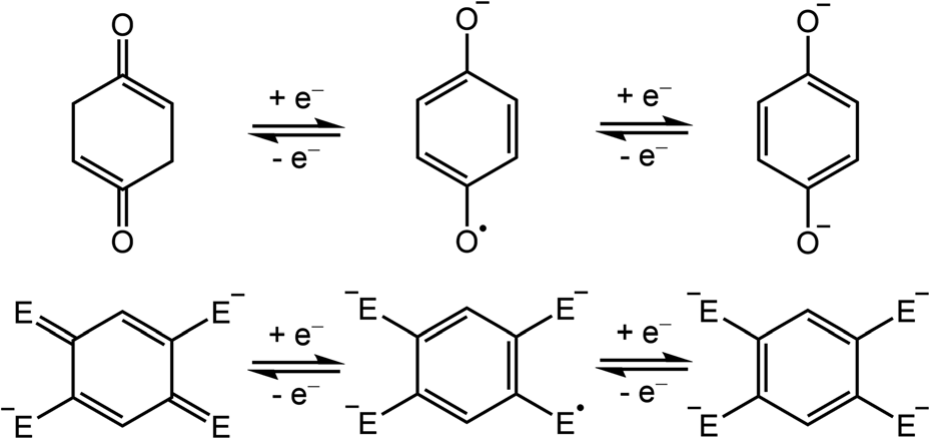
Redox series of bis(monodentate) *p*-quinone (top) and its bis(bidentate) derivatives (bottom; E = O, N*R*, S).

Semiquinone is a ubiquitous free radical in living systems, acting as a radical scavenger^1^ and as a facilitator of electron-transfer processes in photosynthesis^2^ and aerobic respiration.^3^ In these systems, the *p*-quinone molecule serves as an electron reservoir, shuttling between the three consecutive oxidation states of quinone, semiquinone, and hydroquinone (Scheme 1, top). Although the free semiquinone radical cannot be isolated due to its short lifetime, it can be stabilized by coordination to one or more metal centers. Nevertheless, despite the abundance of quinone in living systems, to our knowledge, there are only six studies reporting crystal structures of *p*-semiquinone stabilized by transition metal or lanthanide centers.^4–9^ Of these examples, only one features the *p*-semiquinone radical anion (*p*-C_6_H_4_O_2_˙^−^), as isolated in a structure consisting of two π-stacked radicals coordinated to two Mn^II^(cyclam) units.^8^

In addition to their importance in biological processes, organic radicals have generally garnered much attention as ligands in the design of single-molecule magnets.^10^ In particular, radicals can engage in strong, direct magnetic exchange coupling with metal-based spins, giving rise to energetically well-isolated spin ground states that are necessary to realize slow magnetic relaxation at high temperatures. Semiquinone-based radicals are particularly well-suited toward this end, owing to their negative charge, which enables stronger interactions with metal ions relative to neutral radicals, and their chemical versatility. For instance, 2,5-disubstituted semiquinone derivatives, which can act as bis(bidentate) bridging ligands (Scheme 1, bottom),^11,12^ have been employed to synthesize radical-bridged dinuclear complexes exhibiting strong magnetic exchange coupling^13–16^ and in some cases single-molecule magnet behavior.^17–20^ In contrast, magnetic coupling involving the bis(monodentate) benzosemiquinone molecule (Scheme 1, top) has not been reported.

The use of bis(monodentate) semiquinone as a bridging ligand for magnetic molecules offers several key potential advantages over the more common bis(bidentate) analogues.^11^ First, the presence of only one donor atom per metal ion reduces the overall number of atoms over which the unpaired electron is delocalized, thereby concentrating spin density and promoting stronger exchange. Second, having donor atoms on only two C atoms of the quinone ring leaves the additional four C atoms available for chemical modification. Finally, the occupation of only one metal coordination site by the bridging ligand allows access to metal ions in different coordination geometries. Of particular interest in this regard are trigonal bipyramidal complexes of Ni^2+^, which have been shown to exhibit exceptionally large axial zero-field splitting (*D*) owing to the presence of nearly degenerate d_*x*^2^−*y*^2^_ and d_*xy*_ orbitals.^21,22^ For instance, the compound [(Me_6_tren)NiCl](ClO_4_) (Me_6_tren = tris(2-dimethylaminoethyl)amine) has been shown by high-field, high-frequency electron paramagnetic resonance spectroscopy to exhibit −180 ≤ *D* ≤ −120 cm^−1^.^22^ Despite the potential of [(Me_6_tren)Ni]^2+^ as a building unit for single-molecule magnets, to date no multinuclear molecule featuring this unit has been reported. Moreover, the only example of any complex incorporating multiple trigonal bipyramidal Ni^2+^ centers is a dinuclear imidazole-bridged [Ni(μ-Im)NiL](ClO_4_) complex, which features weak antiferromagnetic coupling between Ni^2+^ centers.^23^ This conspicuous dearth of compounds likely stems from synthetic difficulties associated with the tendency of Ni^2+^ to adopt octahedral coordination.

Herein, we report the synthesis and characterization of the semiquinone-bridged complexes [(Me_6_tren)_2_M^II^_2_(C_6_H_4_O_2_)]^3+^ (M = Fe, Co, Ni), obtained *via* one-electron oxidation of their hydroquinone-bridged precursors. These complexes are the first to feature paramagnetic, trigonal bipyramidal metal centers bridged through a radical ligand and the first examples of any structurally characterized dinuclear complexes bridged through a single semiquinone. Strong, direct magnetic exchange mediated by the semiquinone radical results in well-isolated paramagnetic ground states for all complexes, up to 300 K in the case of Ni. Finally, significant magnetic anisotropy engendered by the trigonal bipyramidal ligand field of the metal centers leads to slow magnetic relaxation under small applied fields, with moderate relaxation barriers for the Co and Ni complexes.

## Results and discussion

### Synthesis and structures

**Scheme 2:**
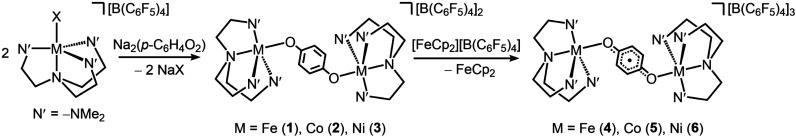
Synthesis of the hydroquinone-bridged compounds [(Me_6_tren)_2_M_2_(C_6_H_4_O_2_^2−^)][B(C_6_F_5_)_4_]_2_ and subsequent chemical oxidation to the semiquinone-bridged compounds [(Me_6_tren)_2_M_2_(C_6_H_4_O_2_^−^˙)][B(C_6_F_5_)_4_]_3_.

The semiquinone-bridged complexes were synthesized in three steps starting from the mononuclear precursors [(Me_6_tren)MX]X (X = Cl^−^ or Br^−^). Initial salt metathesis with K[B(C_6_F_5_)_4_] in acetonitrile afforded [(Me_6_tren)MX][B(C_6_F_5_)_4_] in quantitative yield, and subsequent reaction with Na_2_(*p*-C_6_H_4_O_2_) in tetrahydrofuran (THF) gave the hydroquinone-bridged complexes [(Me_6_tren)_2_M_2_(C_6_H_4_O_2_^2−^)][B(C_6_F_5_)_4_]_2_ (M = Fe (**1**), Co (**2**), Ni (**3**), Scheme 2) in moderate to high yield. Note that the salt metathesis reaction is necessary to generate a THF-soluble precursor, as it was found that coordinating bis(monodentate) semiquinone is displaced by strong donor solvents such as acetonitrile.

Layering Et_2_O onto THF solutions of **1–3** at room temperature afforded large block-shaped crystals of yellow **1**·2THF·Et_2_O, green **2**·2THF·Et_2_O, and dark brown **3**·2THF·Et_2_O, respectively, which were suitable for single-crystal X-ray diffraction analysis. All three compounds crystallize in the space group *P*1̄. Each structure features two metal centers, both in a distorted trigonal bipyramidal geometry with an axial site ligated by an O atom from a bridging dianionic hydroquinone (Fig. S1–S3†). The two metal centers are related through a center of crystallographic inversion symmetry, with M–O–C angles of 124.91(15)°, 131.33(12)°, and 132.08(8)° for Fe, Co, and Ni, respectively (Table 1). Note that **3**·2THF·Et_2_O is only the second example of a multinuclear trigonal bipyramidal Ni^II^ complex,^23^ and it is the first example of any molecule incorporating multiple [(Me_6_tren)Ni]^2+^ units.

**Table 1:** Selected bond distances (Å), angles (°), and structural index, *τ*, obtained for compounds **1–6** at 100 K

	Hydroquinone-bridged complexes			Semiquinone-bridged complexes^a^		
	**1**·2THF·Et_2_O (M = Fe)	**2**·2THF·Et_2_O (M = Co)	**3**·2THF·Et_2_O (M = Ni)	**4**·Et_2_O (M = Fe)	**5**·Et_2_O (M = Co)	**6**·Et_2_O (M = Ni)
C–O	1.319(3)	1.343(2)	1.3405(14)	1.291(4)	1.288(7)	1.296(7)
M–O	1.9019(17)	1.8839(14)	1.8879(9)	1.936(3)	1.922(5)	1.928(4)
M–N_axial_	2.2653(19)	2.2207(15)	2.1207(10)	2.202(3)	2.176(5)	2.070(5)
Mean M–N_eq_	2.2097	2.1583	2.1524	2.179	2.142	2.125
M–O–C	124.91(15)	131.33(12)	132.08(8)	135.1(2)	138.1(4)	136.6(4)
*τ*	0.814	0.837	0.720	0.788	0.820	0.672

a Values are reported from the non-disordered half-molecules in each structure.

In the equatorial plane of each metal center, the average M–N_eq_ bond length decreases by ∼0.05 Å upon moving from Fe to Co to Ni, from 2.2097 to 2.1583 to 2.1524 Å, respectively, in accord with a progressive decrease in metal ionic radius. In contrast, the M–O bond distance decreases by only ∼0.02 Å upon going from Fe to Co to Ni (1.9019(17), 1.8839(14), and 1.8879(9) Å, respectively), which may suggest the greater *trans* influence of the hydroquinone for Fe, and to a lesser extent for Co and Ni. The effect is also reflected in the significant decrease (>0.14 Å) of the M–N_axial_ distance, from 2.2653(19) to 2.2207(15) to 2.1207(10) Å upon moving from Fe to Co to Ni, respectively (Table 1).

Chemical oxidation of **1–3** with one equivalent of [FeCp_2_][B(C_6_F_5_)_4_] afforded the one-electron-oxidized compounds [(Me_6_tren)_2_M_2_(C_6_H_4_O_2_^−^˙)][B(C_6_F_5_)_4_]_3_ (M = Fe (**4**), Co (**5**), Ni (**6**)) in high yield. Surprisingly, these complexes are remarkably air-stable in the solid-state. As monitored by powder X-ray diffraction analysis, **4** shows only slight decomposition after six months, while **5** and **6** maintain crystallinity for an indefinite period of time (Fig. S4–S6†). This kinetic stability may be a result of steric protection of the bridging ligand by the [B(C_6_F_5_)_4_]^−^ counteranions as THF solutions of **4–6** readily decomposed in air.

**Fig. 1:**
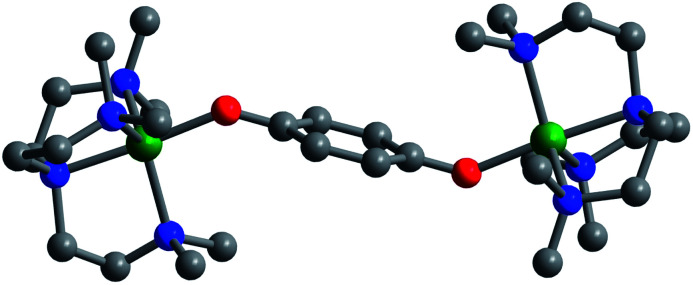
Crystal structure of [(Me_6_tren)_2_Ni_2_(C_6_H_4_O_2_^−^˙)]^3+^, as observed in **6**·Et_2_O collected at 100 K. Green, red, blue, and gray spheres represent Ni, O, N, and C atoms, respectively; H atoms are omitted for clarity.

Layering Et_2_O onto 1,2-difluorobenzene solutions of **4**, **5**, and **6** and storage at −25 °C afforded plate-shaped crystals of dark green **4**·Et_2_O, dark brown **5**·Et_2_O, and red **6**·Et_2_O, which were suitable for single-crystal X-ray diffraction analysis. The three compounds crystallize in the space group *P*2_1_/*c* and are isostructural (Fig. 1 and S7–S9†). In each structure, the asymmetric unit contains two distinct half-molecules of the cationic complex, three [B(C_6_F_5_)_4_]^−^ counterions, and a molecule of Et_2_O. The bond distances and angles associated with the two half-molecules differ considerably in each structure, owing to disorder over two positions for one of the half-molecules. For the purpose of structural comparisons in this report, we refer to the half-molecule without disorder.

The metal centers in **4**, **5**, and **6** retain their trigonal bipyramidal geometry with M–O–C angles of 135.1(2)° (Fe), 138.1(4)° (Co), and 136.6(4)° (Ni), which are slightly greater than those of the unoxidized analogues. Importantly, in contrast to [(cyclam)_2_Mn_2_(C_6_H_4_O_2_^−^˙)_2_](ClO_4_)_2_,^8^ the two M–O axes of the oxidized complexes here adopt a *trans* orientation, which results in a parallel alignment of the M–O vectors. As such, we expect a corresponding parallel alignment of the local magnetic anisotropy axes in **4–6**, as dictated by a crystallographic inversion symmetry of the molecules. Finally, a similar trend in M–N_eq_*vs.* M–O is observed in the oxidized complexes as in the unoxidized congeners described above (Table 1).

A close comparison of bond distances in both series of compounds reveals important information related to the oxidation state of the bridging ligand (Table 1). Upon oxidation, the C–O distances decrease from 1.319(3), 1.343(2), and 1.3405(14) Å, for Fe, Co, and Ni, respectively, to 1.291(4), 1.288(7), and 1.296(7) Å. These changes are consistent with oxidation of the bridging ligand from hydroquinone to semiquinone, which involves a net increase in the C–O bond order. The semiquinone C–O distances in **4**, **5**, and **6** are also consistent with those observed in the structure of [(cyclam)_2_Mn_2_(C_6_H_4_O_2_^−^˙)_2_]^2+^.^8^ The average C–C bond distances appear unchanged within error upon oxidation (Tables S3 and S4†), but these values are complicated by the presence of crystallographic inversion symmetry. Consistent with the weaker donating ability of the monoanionic semiquinone *versus* the dianionic hydroquinone, the M–O bond distance increases upon oxidation, from 1.9019(17), 1.8839(14), and 1.8879(9) Å, for Fe, Co, and Ni, respectively, to 1.936(3), 1.922(5), and 1.928(4) Å. Additionally, the average M–N_eq_ distances change only slightly upon oxidation, from 2.2097, 2.1583, and 2.1524 Å, for Fe, Co, and Ni, respectively, to 2.179, 2.142, and 2.125 Å, further supporting a ligand- rather than metal-based oxidation.

Across the two series of compounds, the Co congeners feature geometries closest to a perfect trigonal bipyramid, as reflected in their *τ* index values of 0.837 (**2**) and 0.820 (**5**), where *τ* = 1 for a perfect trigonal bipyramid and *τ* = 0 for a perfect square pyramid.^24^ This approximate three-fold symmetry for Co^II^ is consistent with a fully symmetric (d_*xz*_,d_*yz*_)^4^(d_*x*^2^−*y*^2^_,d_*xy*_)^2^(d_*z*^2^_)^1^ electronic configuration. In contrast, the Fe^II^ and Ni^II^ complexes bear an odd number of electrons in the (d_*xz*_,d_*yz*_) or (d_*x*^2^−*y*^2^_,d_*xy*_) sets and therefore experience a Jahn–Teller distortion away from three-fold symmetry, as reflected in their lower *τ* indices (Table 1).

### Spectroscopy

**Fig. 2:**
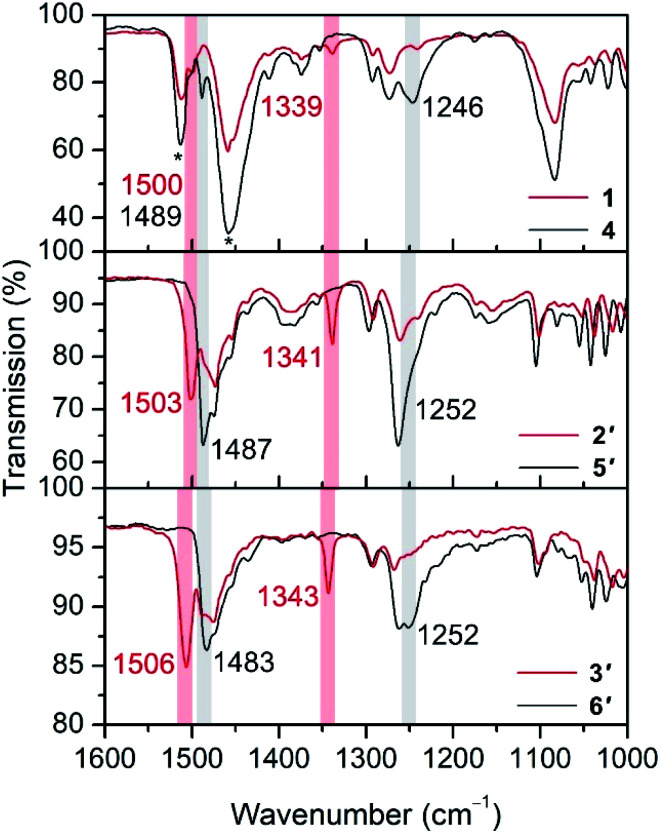
Fourier transform infrared spectra for Fe (top), Co (middle), and Ni (bottom) compounds. Shaded vertical bars and numbers correspond to peaks of interest, as described in the text. Asterisks denote the vibrational modes of [B(C_6_F_5_)_4_]^−^.

The oxidation of hydroquinone in **1–3** to semiquinone in **4–6** is concomitant with a blue shift in the C–O stretching frequency, for instance from 1489 to 1500 cm^−1^ upon oxidation of **1** to **4** (Fig. 2). In the case of the Co and Ni congeners, the C–O stretching frequencies were found to overlap with the vibrational modes of [B(C_6_F_5_)_4_]^−^. We therefore carried out anion exchange reactions for **2**, **3**, **5**, and **6** to access the corresponding PF_6_^−^ salts, denoted as **2′**, **3′**, **5′**, and **6′**, respectively (see the ESI†). The C–O stretches of 1503 and 1506 cm^−1^ in **5′** and **6′**, respectively, are also blue-shifted relative to those for their unoxidized precursors (1487 and 1483 cm^−1^ for **2′** and **3′**, respectively). The C–O vibrational frequencies for all complexes are consistent with values reported previously for hydroquinone and semiquinone coordinated to Mn^II^.^4,8^ In addition, we carried out density functional theory (DFT) calculations on compounds **3** and **6**, which closely reproduce the experimental spectra and support the assignment of the C–O stretching frequencies (Fig. S11†). These calculations also indicate that a second blue-shifted absorption band observed at ∼1340 cm^−1^ for **4**, **5′**, and **6′** corresponds to a C–H rocking mode of semiquinone.

The ^57^Fe Mössbauer spectrum collected for **1** at 5 K features a symmetric Lorentzian doublet with an isomer shift of 1.0320(6) mm s^−1^ and a quadrupole splitting of 3.198(1) mm s^−1^, consistent with a high-spin, five-coordinate Fe^II^ center (Fig. S14†). Upon oxidation, the spectrum for **4** at 20 K is best fit to two similar symmetric Lorentzian doublets, corresponding to the two distinct molecules in the structure, with isomer shifts of 0.996(2) and 1.008(4) mm s^−1^ and quadrupole splittings of 3.528(8) and 3.23(1) mm s^−1^ (Fig. S14†). The minimal decrease in isomer shift observed upon oxidation of **1** to **4** is consistent with a ligand-based oxidation, from hydroquinone to semiquinone, and retention of high-spin Fe^II^ centers.

The UV-visible spectra for **1**, **2**, and **3** feature absorption bands at 314 nm (*ε* = 7 × 10^3^ M^−1^ cm^−1^), 298 nm (*ε* = 3 × 10^3^ M^−1^ cm^−1^), and 355 nm (*ε* = 5 × 10^3^ M^−1^ cm^−1^), respectively (Fig. S17–S20†). These bands are assigned to the hydroquinone π–π* transition, which is red-shifted from the free hydroquinone absorption at 288 nm (*ε* = 2.3 × 10^3^ M^−1^ cm^−1^)^25^ as a result of coordination to the transition metal centers. Several new absorptions were observed in the UV-vis-NIR spectra of **4–6** at longer wavelengths, consistent with transition metal-coordinated semiquinone radical (Fig. S17–S20†),^8,26,27^ and these transitions shift to longer wavelengths upon moving from Ni to Fe (Fig. S20†). Notably, in the case of **4**, these transitions are red-shifted to the NIR region as far as 950 nm.

### Static magnetic properties

**Fig. 3:**
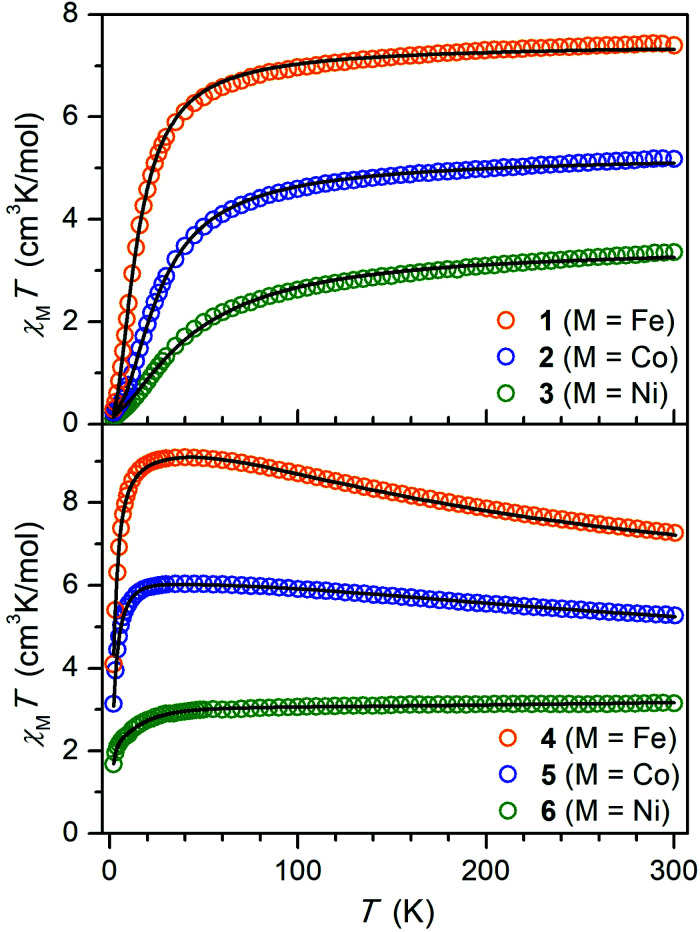
Plots of the molar magnetic susceptibility times temperature (*χ*_M_*T*) *versus T* for **1–3** (top) and **4–6** (bottom) under an applied field of 1 T. Solid lines indicate fits to the data using eqn (1) for **1–3**, eqn (2) for **4** and **5**, and a giant spin approximation with *S* = 3/2 for **6**, as described in the text.

To probe magnetic coupling in the hydroquinone-bridged compounds **1–3**, dc magnetic susceptibility data were collected under an applied field of 1 T, and corresponding plots of *χ*_M_*T vs. T* are shown in the top portion of Fig. 3. At 300 K, *χ*_M_*T* = 7.40, 5.18, and 3.36 cm^3^ K mol^−1^ for **1** (M = Fe), **2** (M = Co), and **3** (M = Ni), respectively, values which are consistent with pairs of magnetically non-interacting *S* = 2 (*g* = 2.22), 3/2 (*g* = 2.35), and 1 (*g* = 2.59) centers. For each complex, the magnitude of *χ*_M_*T* decreases with temperature to minimum values of 0.29 (**1**), 0.22 (**2**), and 0.10 cm^3^ K mol^−1^ (**3**) at 2 K, respectively, which behavior is indicative of weak antiferromagnetic superexchange between the metal centers mediated through the diamagnetic hydroquinone ligand. To quantify the strength of this coupling, the susceptibility data and magnetization data were simultaneously fit according to the Hamiltonian in eqn (1):

1 *Ĥ* = *μ*_B_*H*(***g****Ŝ*_1_ + ***g****Ŝ*_2_) − 2*J*(*Ŝ*_1_·*Ŝ*_2_) + (*Ŝ*_1_·***D***_ion_·*Ŝ*_1_ + *Ŝ*_2_·*D*_ion_·*Ŝ*_2_)

Here, the first, second, and third terms correspond to Zeeman splitting, Heisenberg–Dirac–van Vleck magnetic exchange, and zero-field splitting, respectively, where *S*_1_ and *S*_2_ are the metal-centered spins, ***D***_ion_ is the zero-field splitting tensor for individual metal centers, ***g*** is the *g* tensor of individual metal centers, and *H* is magnetic field. For each complex, ***g*** and ***D***_ion_ for the metal centers were constrained to be equivalent based on the crystallographic inversion symmetry. These fits give exchange constants of *J* = −0.847(2), −2.595(5), and −5.94(1) cm^−1^ for **1**, **2**, and **3**, respectively (Fig. 3, top and Table 2).

**Table 2:** Summary of static magnetic parameters of **1–6**^a^

Compound	M	*g* b	*J* (cm^−1^)	*D*_ion_ c (cm^−1^)
**1**	Fe	2.23	−0.847(2)	−10.48(5)
**2**	Co	2.42	−2.595(5)	−24.4(1)
**3**	Ni	2.72	−5.94(1)	−96.6(6)
**4**	Fe	2.13	−144(1)	7.78(5)
**5**	Co	2.28	−252(2)	10.9(1)
**6**	Ni		<−600	

a Obtained by fitting or simulating dc susceptibility data for **1–5** and **6**, respectively, as described in the text.

b Isotropic *g* value as calculated from averaging the anisotropic ***g*** tensor (Table S7).

c The axial component of the ***D***_ion_ tensor.

The semiquinone-bridged complexes of compounds **4** (M = Fe), **5** (M = Co), and **6** (M = Ni) exhibit starkly different magnetic behavior. In the case of **4** and **5**, the *χ*_M_*T* curves increase steadily with decreasing temperature to reach maximum values of 9.11 and 6.04 cm^3^ K mol^−1^ at 45 K, respectively, before undergoing subsequent downturns. In the case of **6**, *χ*_M_*T* decreases only slightly in a linear fashion with temperature from 3.15 cm^3^ K mol^−1^ at 300 K to 3.00 cm^3^ K mol^−1^ at 50 K, characteristic of temperature-independent paramagnetism, before dropping more precipitously below 50 K to a value of 1.68 cm^3^ K mol^−1^ at 2 K. For each complex, this behavior is indicative of strong antiferromagnetic coupling between the semiquinone radical and the two metal centers as well as the presence of significant zero-field splitting. To quantify the magnetic coupling, the susceptibility and magnetization data for **4** and **5** were fit using the Hamiltonian given by eqn (2):

2 *Ĥ* = *μ*_B_*H*(***g****Ŝ*_1_ + ***g****Ŝ*_2_ + ***g***_SQ_*Ŝ*_SQ_) − 2*J*(*Ŝ*_1_·*Ŝ*_SQ_ + *Ŝ*_2_·*Ŝ*_SQ_) + (*Ŝ*_1_·***D***_ion_·*Ŝ*_1_ + *Ŝ*_2_·***D***_ion_·*Ŝ*_2_)

Here, *S*_SQ_ = 1/2 represents the spin of the semiquinone radical. As above, ***D***_ion_ and ***g*** for both metal centers in each complex were constrained to be the same due to the crystallographic inversion symmetry, and *g*_SQ_ was constrained to be 2.00. These fits afforded exchange constants of *J* = −144(1) and −252(2) cm^−1^ for **4** and **5**, respectively (Fig. 3, bottom and Table 2).

The *χ*_M_*T vs. T* data for **6** could not be satisfactorily fit to eqn (2). Nevertheless, simulation of the data revealed the presence of very strong antiferromagnetic coupling with an estimated upper bound of *J* < −600 cm^−1^. In line with this result, broken-symmetry DFT calculations carried out using the solid-state structure of **6** predicted an exchange constant of *J* = −542 cm^−1^ (see the ESI for details†). Given this large magnitude of *J*, the *S* = 3/2 ground state in **6** should be well-isolated from excited states even at 300 K, and indeed the magnetic susceptibility data could be fit using a giant spin approximation with *S* = 3/2 (Fig. 3, bottom). A simultaneous fit of the susceptibility and low-temperature magnetization data (Fig. S26†) for *S* = 3/2 yielded the following parameters: *g*_∥_ = 2.781(2), *g*_⊥_ = 2.357(3), *D*_mol_ = −21.4(2) cm^−1^, *E*_mol_ = 4.6(2) cm^−1^, and *χ*_TIP_ = 6.6(2) × 10^−4^ cm^3^ mol^−1^ (Table 3). Note that **6** represents the first multinuclear Ni complex with a thermally isolated magnetically coupled ground state at 300 K.

**Table 3:** Zero-field splitting parameters and corresponding calculated spin reversal barriers (*U*_calc_) compared with experimental spin reversal barriers (*U*_eff_) extracted from dynamic magnetic data for semiquinone-bridged compounds **4** (M = Fe), **5** (M = Co), and **6** (M = Ni)

Compound	*S*	*g*_∥_	*g*_⊥_	*D*_mol_ (cm^−1^)	|*E*_mol_| (cm^−1^)	*U*_calc_ (cm^−1^)	*U*_eff_ (cm^−1^)	*τ*_0_ (ns)
**4**	7/2	1.976(7)	2.2002(9)	3.95(3)	0.579(4)	12.1	—	—
**5**	5/2	1.962(4)	2.576(1)	6.18(3)	1.55(2)	18.4	22.0(1)	2.6(1)
**6**	3/2	2.781(2)	2.357(3)	−21.4(2)	4.6(2)	45.6	45.9(4)	0.40(4)

The magnetic data obtained for **4–6** are the first reported for any *p*-semiquinone-bridged transition metal complex, and they demonstrate that the semiquinone radical can facilitate extremely strong magnetic coupling *via* direct exchange through the ligand-based unpaired electron. Indeed, the magnitudes of *J* found here are much larger than those mediated *via* most radical ligands,‡‡A few exceptions include [(Co(tpy))_2_(tphz)]^3+^ which exhibits a well-isolated *S* = 5/2 ground state^39^ and [K(18-c-6)][(Co(N(SiMe_3_)_2_)_2_)_3_-(HAN)] (*J*_Co-rad_ = −290 cm^−1^).^40^ such as tetrazine (+96 cm^−1^ with Ni^2+^),^28^ nindigo (−138 cm^−1^ with Co^2+^),^29^ and phenazine (+149 cm^−1^ with Ni^2+^).^30^ Further, the coupling observed for **4** and **5** is considerably stronger than that previously reported for bis(bidentate) 2,5-dihydroxy-1,4-benzoquinone derivatives with Fe (*J* = −57 to −65 cm^−1^)^20^ and Co (*J* = −52 cm^−1^).^15^ Likely, this prominent difference results predominantly from the delocalization of the unpaired electron over only two O atoms in semiquinone *versus* four in the bis(bidentate) analogues. In addition, it is possible that the presence of fewer electronegative O atoms increases the energy of the singly occupied molecular orbital of the semiquinone, thus resulting in a better energy match with the metal orbitals. In fact, the coupling constant for **4** is comparable to that determined for the *o*-semiquinone radical in the mononuclear [PhTt^tBu^]Fe(phenSQ) complex (*J*_Fe–L_ = −127 cm^−1^).^31^ Nevertheless, stronger exchange has been observed for semiquinonoid ligands with N-donors (*J* < −900 cm^−1^ with Fe^2+^ and −440 cm^−1^ with Co^2+^),^17,19^ as expected for a more diffuse, higher-energy SOMO of N compared to O.^16,32^ As such, incorporation of bridging ligands based on *p*-phenylenediamine radical may provide a route to complexes with even stronger coupling.

Below 20 K, the magnetization data for **4**, **5**, and **6** were fit using a giant spin Hamiltonian with values of *S* = 7/2, 5/2, and 3/2, respectively (Fig. S24–S26†), and the corresponding zero-field splitting parameters are listed in Table 3. With these zero-field splitting parameters, the program PHI^33^ was used to calculate ground to first excited state splittings (*U*_calc_) of 12.1, 18.4, and 45.6 cm^−1^ for **4**, **5**, and **6**, respectively (Table 3).

### Dynamic magnetic properties

The static magnetization data for the semiquinone-bridged compounds **4–6** indicate magnetic ground states with significant anisotropy and moderate excited state splittings, suitable for the observation of single-molecule magnet behavior. We thus set out to probe the spin dynamics of each compound *via* ac magnetic susceptibility measurements. Under a 1000 Oe field, the Fe^II^-containing compound **4** exhibits tails in the out-of-phase molar magnetic susceptibility 
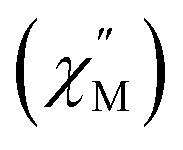
 at the low and high frequency limits of the magnetometer (Fig. S27†). The slow process rapidly diminishes to zero with increasing temperature, which suggests it may be dipolar in origin,^34^ and the fast process moves out of the magnetometer range with increasing temperature.

**Fig. 4:**
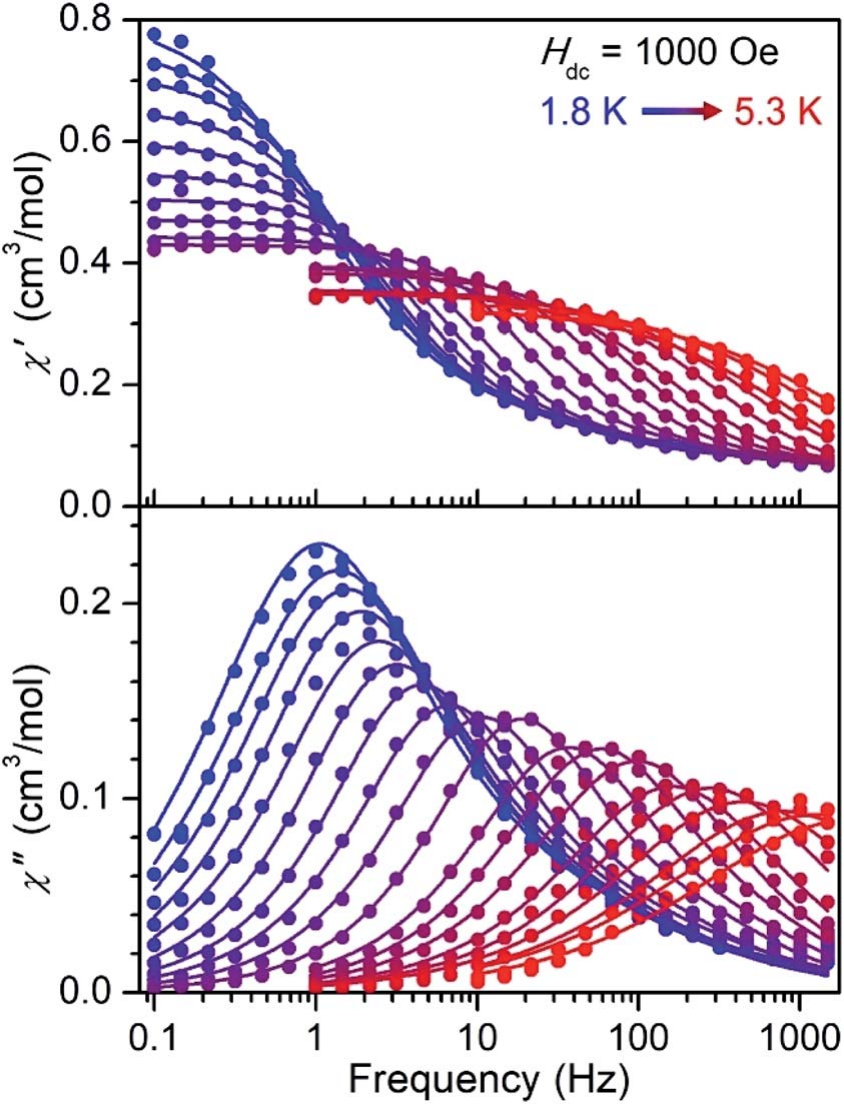
Molar in-phase (top) and out-of-phase (bottom) magnetic susceptibility *versus* frequency data for compound **6** measured under a 1000 Oe field from 1.8 to 5.3 K. Data are represented by colored symbols and solid lines represent fits to the data using a generalized Debye model^33^ with two relaxation processes.

The Co^II^-containing compound **5** shows slow magnetic relaxation under zero applied field that is further slowed under dc fields (Fig. S29†). Under an optimal field of 2000 Oe, **5** exhibits two peaks in the out-of-phase susceptibility from 1.8–3.0 K, one below 1 Hz that can be ascribed to dipolar interactions^34^ and a thermally activated process at higher frequencies (Fig. S31†). Similarly, under a 1000 Oe field, the Ni^II^-containing compound **6** exhibits a single peak in 
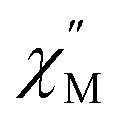
 from 1.8 to 5.3 K (Fig. 4), and the data feature slight asymmetry that might arise due to the disordered second molecule in the structure (see the ESI†). The ac magnetic susceptibility data for **5** and **6** were fit using a generalized Debye model^35^ with two relaxation processes (see the ESI for details†). In the case of **6**, the relaxation is dominated by a single major component (Table S9 and Fig. S36†), which is the focus of further discussion below.

**Fig. 5:**
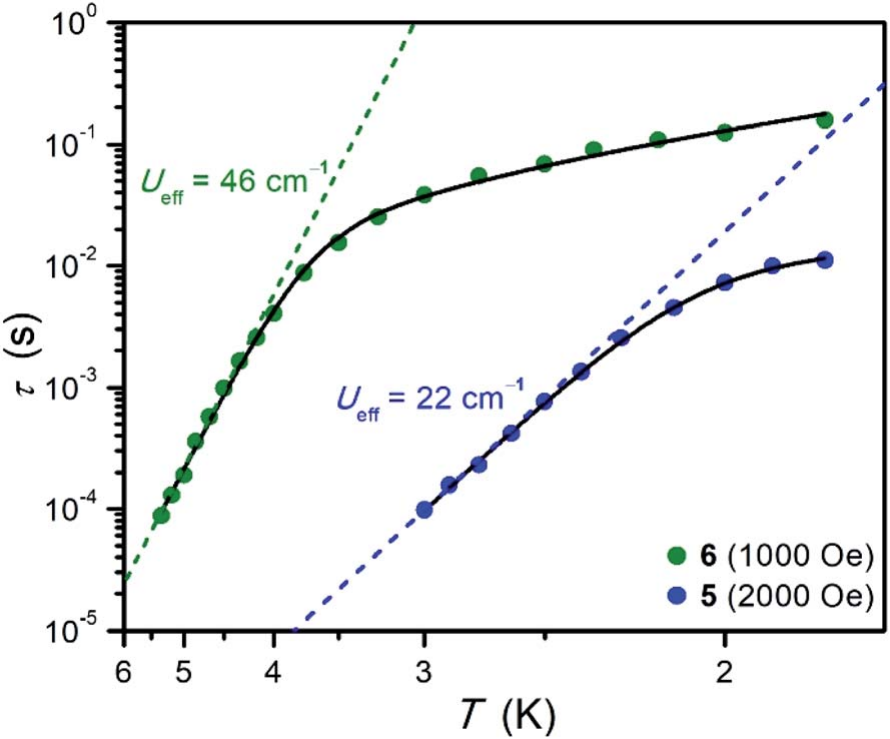
Arrhenius plots of relaxation time for **5** and **6**. Data were collected under an optimized dc field of 2000 Oe for **5** and 1000 Oe for **6**. Black lines represent fits to the data according to eqn (3), and dashed lines represent the contribution from Orbach relaxation. The error in *τ* is smaller than the height of the corresponding symbols.

Temperature-dependent relaxation times were extracted for **5** and **6** and are plotted *versus T* in Fig. 5. The Arrhenius plot for each compound exhibits a linear region at high temperatures, which is indicative of spin relaxation through an Orbach process. The temperature-dependent relaxation data were fit to eqn (3):3
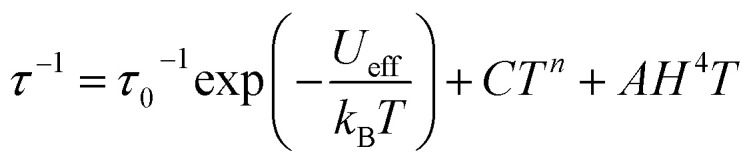
where the first, second, and third terms correspond to Orbach, Raman, and direct processes, respectively. In this expression, *τ*_0_ is a pre-exponential factor, *U*_eff_ is the effective spin relaxation barrier, *k*_B_ is the Boltzmann constant, *T* is temperature, *C* and *A* are constants for the Raman and direct processes, respectively, *n* is the Raman exponent (typically 3–9) and *H* is magnetic field.^36,37^ The data for **5** could be fit without the Raman term, yielding *U*_eff_ = 22.0(1) cm^−1^, *τ*_0_ = 2.6(1) × 10^−9^ s, and *A* = 2.67(5) × 10^4^ s^−1^ T^−4^ K^−1^. The data for **6** were fit without inclusion of the direct process term, yielding *U*_eff_ = 45.9(4) cm^−1^, *τ*_0_ = 4.0(4) × 10^−10^ s, *C* = 0.97(3) s^−1^ K^−3^, and *n* = 3. Note that the suitability of this model for the relaxation dynamics, particularly at higher temperatures, is supported by the *τ*_0_ values in the expected range for Orbach relaxation^35^ and the similarity between the *U*_eff_ and *U*_calc_ values determined from static magnetization measurements for both compounds (Table 3).

The Orbach relaxation observed for **6** is in remarkable contrast to that observed for mononuclear Ni^II^ complexes in similar coordination environments. For example, the trigonal bipyramidal complex in [Ni(MDABCO)_2_Cl_3_]ClO_4_ exhibits very fast relaxation dominated by a Raman process,^21^ and the compound Li(THF)[Ni((Me_3_SiNCH_2_CH_2_)_3_N)] does not show slow magnetic relaxation at all.^38^ Moreover, previous EPR characterization of the compounds [(Me_6_tren)NiCl]ClO_4_ and [(Me_6_tren)NiBr]Br revealed the presence of extremely large values of *D*, yet no evidence of slow magnetic relaxation was reported.^22^ Note that the only previously reported example of a complex with multiple trigonal bipyramidal nickel ions is an imidazole-bridged Ni_2_ complex, which features a non-magnetic ground state.^23^ Based on these precedents, we postulate that the strong magnetic exchange through the semiquinone bridge in **6** serves to limit fast relaxation *via* Raman and direct processes and thus enables observation of Orbach relaxation. Our results support a growing number of studies that demonstrate the utility of judiciously selected multinuclear single-molecule magnets featuring radical-bridged, high-anisotropy metal ions for suppressing through-barrier relaxation processes.^17–20^

## Conclusions

The foregoing results show that semiquinone can be employed as a bridging ligand between metal ions to give exceptionally strong metal–radical magnetic coupling and single-molecule magnet behavior in [(Me_6_tren)_2_M_2_(C_6_H_4_O_2_^−^˙)]^3+^ (M = Fe, Co, Ni). Magnetic susceptibility data obtained for these complexes reveal the presence of strong metal–semiquinone coupling, with exchange constants of *J* = −144(1) and −252(2) cm^−1^ for Fe and Co, respectively, and *J* < −600 cm^−1^ for Ni. Importantly, the values of *J* for Fe and Co are substantially larger in magnitude than those reported for related bis(bidentate) semiquinoid complexes, demonstrating that decreasing the number of donor atoms can increase magnetic coupling strength. Additionally, the enhanced magnetic exchange achieved with semiquinone results in the first multinuclear Ni complex with a thermally isolated ground state. The presence of field-induced molecular slow magnetic relaxation for the Co and Ni semiquinone-bridged complexes further serves to illustrate the efficacy of coupling anisotropic metal centers to engender single-molecule magnet behavior. Current efforts are underway to incorporate semiquinone bridges into molecules with heavier metal ions and into extended networks.

## Conflicts of interest

There are no conflicts to declare.

## Supplementary Material

SC-011-D0SC03078C-s001

SC-011-D0SC03078C-s002
