# Supplementary material for: Semiquinone radical-bridged M2 (M = Fe, Co, Ni) complexes with strong magnetic exchange giving rise to slow magnetic relaxation
Source: Chem Sci. 2020 Jul 21;11(31):8196–203. doi: 10.1039/d0sc03078c (PMC8163326; doi:10.1039/d0sc03078c)
Supplement: SC-011-D0SC03078C-s001 [file SC-011-D0SC03078C-s001.pdf]

Electronic Supplementary Material (ESI) for Chemical Science.
This journal is © The Royal Society of Chemistry 2020

Electronic Supplementary Information for

Semiquinone Radical-Bridged M_2 (M = Fe, Co, Ni) Complexes with Strong Magnetic Exchange Giving Rise to Slow Magnetic Relaxation

Khetpakorn Chakarawet,[1] T. David Harris,[1] and Jeffrey R. Long[*,1,2,3]

[1]Department of Chemistry, University of California Berkeley, Berkeley, California 94720, United States

[2]Department of Chemical and Biomolecular Engineering, University of California Berkeley, Berkeley, California 94720, United States

[3]Materials Sciences Division, Lawrence Berkeley National Laboratory, Berkeley, California 94720, United States

*correspondence to: jrlong@berkeley.edu

Table of Contents **Page**

Experimental Details	S2
X-ray Crystallography Data Collection and Refinement Details	S5
Density Functional Theory Calculations	S16
Spectroscopy	S18
Magnetic Measurements	S25
References	S42

Experimental Details

General Procedures. All air-sensitive chemistry was performed in a glovebox under an inert atmosphere of Ar or on a Schlenk line manifold filled with Ar. Prior to use, tetrahydrofuran (THF), acetonitrile, hexane, Et_2O, and 1,2-difluorobenzene (DFB) were deoxygenated and dried using a commercial solvent purification system designed by JC Meyer Solvent Systems and then stored over 4 Å molecular sieves. Hydroquinone was purchased from Sigma-Aldrich and sublimed before use. Tetra-*n*-butylammonium hexafluorophosphate was purchased from Sigma-Aldrich and used as received. The compounds [Fe(Me_6tren)Br]Br, [Co(Me_6tren)Cl]Cl, [Ni(Me_6tren)Br]Br,[1,2] and [$FeCp_2$][$B(C_6F_5)_4$][3] were synthesized according to literature procedures. Elemental analyses were performed by the Microanalytical Laboratory at the University of California, Berkeley, using a Perkin Elmer 2400 Series II combustion analyzer.

Na_2(*p*-$C_6H_4O_2$)·0.7THF. In a glovebox, a solid mixture of *p*-hydroquinone (0.4111 g, 3.734 mmol) and NaH (0.1794 g, 7.475 mmol) was charged with THF (10 mL). The reaction mixture was stirred for 18 h to yield a white slurry. The solid was isolated on a medium porosity frit, washed with THF (3 × 3 mL), and dried under reduced pressure to yield the desired product as free-flowing white powder (0.7382 g, 3.609 mmol, 97%).

Elem. Anal. Found: C 51.48, H 4.93%. Calcd for Na_2(*p*-$C_6H_4O_2$)·0.7THF ($Na_2O_{2.7}C_{8.8}H_{9.6}$): C 51.67, H 4.73%.

[Fe(Me_6tren)Br][$B(C_6F_5)_4$]. In a glovebox, a solid mixture of [Fe(Me_6tren)Br]Br (0.2980 g, 0.6681 mmol) and K[$B(C_6F_5)_4$] (0.4832 g, 0.6728 mmol) was charged with acetonitrile (8 mL). A white solid precipitated from the transparent, colorless mixture immediately. The reaction mixture was stirred for 1 h. The solvent was then removed under reduced pressure, and the ensuing residue was washed with Et_2O (3 × 2 mL). The remaining solid was dissolved in THF (5 mL), filtered through glass microfiber, and dried under reduced pressure to yield the desired product as an off-white crystalline solid in quantitative yield (0.701 g, 0.671 mmol, 100%). The material was deemed sufficiently pure for subsequent synthesis and its purity was not further characterized.

[Co(Me_6tren)Cl][$B(C_6F_5)_4$]. Under air, a solid mixture of [Co(Me_6tren)Cl]Cl (0.4895 g, 1.359 mmol) and K[$B(C_6F_5)_4$] (0.9740 g, 1.356 mmol) was charged with acetonitrile (10 mL). A white solid precipitated from the blue mixture immediately. The reaction mixture was stirred for 1 h. The solvent was then removed under reduced pressure, and the ensuing residue was washed with Et_2O (3 × 5 mL). The remaining solid was dissolved in THF (10 mL), filtered through glass microfiber, and dried under reduced pressure to yield the desired product as blue crystalline solid in quantitative yield (1.358 g, 1.353 mmol, 99.8%). The material was deemed sufficiently pure for subsequent synthesis and its purity was not further characterized.

[Ni(Me_6tren)Br][$B(C_6F_5)_4$]. Under air, a solid mixture of [Ni(Me_6tren)Br]Br (0.7006 g, 1.561 mmol) and K[$B(C_6F_5)_4$] (1.1194 g, 1.559 mmol) was charged with acetonitrile (15 mL). A white solid precipitated from the yellow aliquot immediately. The reaction mixture was stirred for 1 h. The solvent was then removed under reduced pressure, and the ensuing residue was washed with Et_2O (3 × 5 mL). The remaining solid was dissolved in THF (10 mL), filtered through glass microfiber, and dried under reduced pressure to yield the desired product as yellow crystalline

solid in quantitative yield (1.644 g, 1.569 mmol, 100%). The material was deemed sufficiently pure for subsequent synthesis and its purity was not further characterized.

$[(Me_6tren)_2Fe_2(C_6H_4O_2)][B(C_6F_5)_4]_2$ (1). In a glovebox, a solid mixture of $[Fe(Me_6tren)Br][B(C_6F_5)_4]$ (0.3026 g, 0.2895 mmol) and $Na_2(p\text{-}C_6H_4O_2)\cdot0.7THF$ (0.0450 g, 0.220 mmol) was charged with THF (6 mL). The reaction mixture was stirred for 24 h, during which time the reaction mixture became a yellow suspension. The solvent was removed under reduced pressure. The product was washed with Et_2O (3 × 2 mL), extracted with THF, filtered through a bed of diatomaceous earth, and dried under reduced pressure to yield **1** as yellow powder (0.2703 g, 0.1326 mmol, 92%). Large yellow block crystals of **1**·2THF·Et_2O (0.2294 g, 0.1016 mmol, 70%) formed after 2 d from a THF solution of **1** layered with Et_2O at room temperature.

Elem. Anal. Found: C 48.31, H 4.18, N 5.13%. Calcd for **1**·2THF·Et_2O ($Fe_2F_{40}O_5N_8C_{90}B_2H_{90}$): C 47.89, H 4.02, N 4.96%.

$[(Me_6tren)_2Co_2(C_6H_4O_2)][B(C_6F_5)_4]_2$ (2). In a glovebox, a solid mixture of $[Co(Me_6tren)Cl][B(C_6F_5)_4]$ (0.3038 g, 0.3026 mmol) and $Na_2(p\text{-}C_6H_4O_2)\cdot0.7THF$ (0.0602 g, 0.294 mmol) was charged with THF (8 mL). The reaction mixture was stirred for 23 h, during which time the reaction mixture became a blue-green suspension. The solvent was removed under reduced pressure. The product was washed with Et_2O (3×2 mL), extracted with THF, filtered through a bed of diatomaceous earth, and dried under reduced pressure to yield **2** as green powder (0.2487 g, 0.1216 mmol, 80%). Large green block crystals of **2**·2THF·Et_2O (0.2146 g, 0.0948 mmol, 63%) formed after 2 d from a THF solution of **2** layered with Et_2O at room temperature.

Elem. Anal. Found: C 47.56, H 3.65, N 5.05%. Calcd for **2**·2THF·Et_2O ($Co_2F_{40}O_5N_8C_{90}B_2H_{90}$): C 47.76, H 4.01, N 4.95%.

$[(Me_6tren)_2Ni_2(C_6H_4O_2)][B(C_6F_5)_4]_2$ (3). In a glovebox, a solid mixture of $[Ni(Me_6tren)Br][B(C_6F_5)_4]$ (0.2889 g, 0.276 mmol) and $Na_2(p\text{-}C_6H_4O_2)\cdot0.7THF$ (0.0532 g, 0.260 mmol) was charged with THF (7 mL). The reaction mixture was stirred for 24 h, during which time the reaction mixture became a dark brown suspension. The solvent was removed under reduced pressure. The product was washed with Et_2O (3×2 mL), extracted with THF, filtered through a bed of diatomaceous earth, and dried under reduced pressure to yield **3** as dark brown solid (0.2730 g, 0.134 mmol, 97%). Large dark brown crystals of **3**·2THF·Et_2O (0.1613 g, 0.0721 mmol, 52%) formed after 2 d from a THF solution of **3** layered with Et_2O at room temperature.

Elem. Anal. Found: C 47.69, H 3.88, N 4.95%. Calcd for **3**·2THF·Et_2O ($Ni_2F_{40}O_5N_8C_{90}B_2H_{90}$): C 47.77, H 4.01, N 4.95%.

$[(Me_6tren)_2Fe_2(C_6H_4O_2)][B(C_6F_5)_4]_3$ (4). In a glovebox, a solid mixture of **1** (0.0733 g, 0.0360 mmol) and $[FeCp_2][B(C_6F_5)_4]$ (0.0309 g, 0.0357 mmol) was charged with THF (4 mL). The reaction mixture immediately turned dark green. The mixture was stirred for 30 min, and the solvent was removed under reduced pressure. The product was washed with Et_2O (3×2 mL) to remove $FeCp_2$ byproduct as yellow solution. The product was dried under reduced pressure to afford **1** as dark green powder (0.0954 g, 0.0351 mmol, 98%). The product was then dissolved in DFB, layered with Et_2O, and stored for 3 d at −25 °C to yield **4**·Et_2O as dark green plate crystals.

Elem. Anal. Found: C 45.42, H 2.91, N 3.82%. Calcd for **4**·Et_2O ($Fe_2F_{60}O_3N_8C_{106}B_3H_{74}$): C 45.60, H 2.67, N 4.01%.

[(Me_6tren)$_2Co_2$($C_6H_4O_2$)][B(C_6F_5)$_4$]$_3$ (5). In a glovebox, a solid mixture of **2**·2THF·Et_2O (0.0468 g, 0.0229 mmol) and [$FeCp_2$][B(C_6F_5)$_4$] (0.0203 g, 0.0235 mmol) was charged with THF (2 mL). The reaction mixture immediately turned dark brown. The mixture was stirred for 15 min, and the solvent was then removed under reduced pressure. The product was washed with Et_2O (4×1 mL) to remove $FeCp_2$ byproduct as yellow solution. The product was dried under reduced pressure to afford **2** as dark brown solid (0.579 g, 0.0213 mmol, 93%). The product was crystallized by layering Et_2O onto a DFB solution of the compound and storing at –25 °C for 3 d, yielding **5**·Et_2O as dark brown plates.

Elem. Anal. Found: C 45.35, H 2.56, N 4.32%. Calcd for **5**·Et_2O ($Co_2F_{60}O_3N_8C_{106}B_3H_{74}$): C 45.50, H 2.67, N 4.00%.

[(Me_6tren)$_2Ni_2$(*p*-$C_6H_4O_2$)][B(C_6F_5)$_4$]$_3$ (6). In a glovebox, a solid mixture of **3**·2THF·Et_2O (0.1030 g, 0.0455 mmol) and [$FeCp_2$][B(C_6F_5)$_4$] (0.0396 g, 0.0458 mmol) was charged with THF (5 mL). The reaction mixture immediately turned intensely red. The mixture was stirred for 1 h, and the solvent was removed under reduced pressure. The product was washed with hexane (2 mL) and Et_2O (3×2 mL) to remove $FeCp_2$ byproduct as yellow solution. The product was dried under reduced pressure to afford **6** as bright red solid (0.1124 g, 0.0413 mmol, 91%). The product was crystallized after 3 d from DFB solution layered with Et_2O and stored at –25 °C, yielding **6**·Et_2O as red plates.

Elem. Anal. Found: C 45.86, H 2.62, N 3.90%. Calcd for **6**·Et_2O ($Ni_2F_{60}O_3N_8C_{106}B_3H_{74}$): C 45.51, H 2.67, N 4.01%.

Anion exchange of [(Me_6tren)$_2M_2$($C_6H_4O_2$)][B(C_6F_5)$_4$]$_n$ with $(PF_6)^-$ (M = Co, Ni; *n* = 2, 3)

A mixture of [(Me_6tren)$_2M_2$($C_6H_4O_2$)][B(C_6F_5)$_4$]$_n$ (M = Co or Ni, *n* = 2 or 3) (0.003 mmol) and excess [N^nBu_4](PF_6) (0.030 mmol) was charged with THF (1 mL). The reaction mixture was stirred for 10 min. The resulting precipitate was washed with THF and dried to yield the desired [(Me_6tren)$_2M_2$($C_6H_4O_2$)](PF_6)$_n$ salt (**2′**: M = Co, *n* = 2; **3′**: M = Ni, *n* =2; **5′**: M = Co, *n* = 3; **6′**: M = Ni, *n* = 3), which was used to obtain FTIR spectra described in the main text (see Figure 2).

X-ray Crystallography Data Collection and Refinement Details

Large block crystals of **1**·2THF·Et_2O (yellow), **2**·2THF·Et_2O (green), and **3**·2THF·Et_2O (dark brown) suitable for single-crystal X-ray diffraction were grown from a mixture of THF and Et_2O.

Dark green (**4**), dark brown (**5**) and intense red (**6**) crystals were grown from a mixture of DFB and Et_2O. Crystals grown in this mixed solvent system only diffracted to 0.88 Å for **4**, 0.93 Å for **5**, and 1.03 Å for **6**, using synchrotron radiation. Several crystallization setups were attempted, but the resolution could not be improved. Other solvent combinations did not yield any crystals, nor did exchanging anions yield any crystalline solid upon crystallization.

Single-crystal X-ray diffraction data were collected at Beamline 12.2.1 at the Advanced Light Source, Lawrence Berkeley National Laboratory using synchrotron radiation (λ = 0.7288 Å). Single crystals were coated with Paratone-N oil, mounted on a MiTeGen loop, and frozen at 100 K under a N_2 stream of an Oxford Cryosystems Cryostream 700 Plus. The X-ray intensity data were measured using a Bruker SMART Apex II diffractometer. Data were collected from four 180° ω scans with 1° steps at 90° rotation intervals about φ and a CCD detector distance of 5 cm. The frames were integrated with the Bruker SAINT Software package using a narrow-frame algorithm.[4] Absorption correction was performed through SADABS.[5] Structure solutions were performed by SHELXT[6] using the direct method and were refined by least-square refinement against F^2 by SHELXL[7] following standard procedures through the integrated structure analysis program OLEX2.[8]

[(FeMe$_6$tren)$_2$(*p*-$C_6H_4O_2$)][B(C_6F_5)$_4$]$_2$·2THF·Et_2O (1·2THF·Et_2O). All non-H atoms were refined anisotropically. H atoms were placed on geometrically calculated positions using riding model. A THF molecule was found in the structure and was found to be disordered over two positions. Anisotropic refinement of the disordered THF molecule was stabilized by application of enhanced rigid bond restraints. There is a solvent-accessible void which was not successfully modeled due to a highly disordered solvent molecule. The structure of the isostructural [(NiMe$_6$tren)$_2$(*p*-$C_6H_4O_2$)][B(C_6F_5)$_4$]$_2$ (**3**) was successfully modeled, indicating that this space is occupied by a Et_2O molecule disordered over a crystallographic inversion center. In the structure of **1**, the disorder was not successfully modeled, and SQUEEZE command was used.

[(CoMe$_6$tren)$_2$(*p*-$C_6H_4O_2$)][B(C_6F_5)$_4$]$_2$·2THF·Et_2O (2·2THF·Et_2O). All non-H atoms were refined anisotropically. H atoms were placed on geometrically calculated positions using riding model. A THF molecule was found in the structure and was found to be disordered over two positions. Anisotropic refinement of the disordered THF molecule was stabilized by application of enhanced rigid bond restraints. There is a solvent-accessible void which was not successfully modeled due to a highly disordered solvent molecule. The structure of the isostructural [(NiMe$_6$tren)$_2$(*p*-$C_6H_4O_2$)][B(C_6F_5)$_4$]$_2$ (**3**) was successfully modeled, indicating that this space is occupied by a Et_2O molecule disordered over a crystallographic inversion center. In the structure of **2**, the disorder was not successfully modeled, and SQUEEZE command was used.

[(NiMe$_6$tren)$_2$(*p*-$C_6H_4O_2$)][B(C_6F_5)$_4$]$_2$·2THF·Et_2O (3·2THF·Et_2O). All non-H atoms were refined anisotropically. H atoms were placed on geometrically calculated positions using riding model. A Et_2O molecule was found to be disordered over a crystallographic inversion center, and

anisotropic refinement was stabilized by the enhanced rigid bond restraints. In addition, the C–O and C–C distances were restrained to the appropriate values. A THF molecule was found in the structure and was found to be disordered over two positions. Refinement of the THF molecule did not require any ADP restraint.

[(FeMe$_6$tren)$_2$(*p*-C$_6$H$_4$O$_2$)][B(C$_6$F$_5$)$_4$]$_3$·Et$_2$O (4·Et$_2$O). Compound **4** crystallized as dark green plate crystals. The crystals diffracted poorly, partly because of the plate morphology. After several attempts, the best crystal was chosen for data collection. Good quality data were acquired down to 0.88 Å. Despite poor resolution, refinement of the structure required minimal restraints. Two half-molecules of $[(FeMe_6tren)_2(C_6H_4O_2)]^{3+}$, along with three $[B(C_6F_5)_4]^-$ anions, were found in the asymmetric unit. The remaining half of the molecule were generated by an inversion symmetry. The second molecule of $[(FeMe_6tren)_2(C_6H_4O_2)]^{3+}$ was found to be disordered by the Me$_6$tren ligand and was modeled over two positions. Anisotropic refinement of the disordered Me$_6$tren ligand was stabilized by the enhanced rigid bond restraints. All non-H atoms were refined anisotropically. All H atoms were placed on the geometrically calculated positions using the riding model.

[(CoMe$_6$tren)$_2$(*p*-C$_6$H$_4$O$_2$)][B(C$_6$F$_5$)$_4$]$_3$·Et$_2$O (5·Et$_2$O). Compound **5** crystallized as brown plate crystals. The crystals diffracted poorly, partly because of the plate morphology. After several attempts, the best crystal was chosen for data collection. Good quality data were acquired down to 0.93 Å. Despite poor resolution, refinement of the structure required minimal restraints. Two half-molecules of $[(CoMe_6tren)_2(C_6H_4O_2)]^{3+}$, along with three $[B(C_6F_5)_4]^-$ anions, were found in the asymmetric unit. The remaining half of the molecule were generated by an inversion symmetry. The second molecule of $[(CoMe_6tren)_2(C_6H_4O_2)]^{3+}$ was found to be disordered by the Me$_6$tren ligand and was modeled over two positions. The disordered Co2–N7 and Co2–N7A distances were restrained to be the same. Anisotropic refinement of the disordered Me$_6$tren ligand was stabilized by the enhanced rigid bond restraints. All non-H atoms were refined anisotropically. All H atoms were placed on the geometrically calculated positions using the riding model.

[(NiMe$_6$tren)$_2$(*p*-C$_6$H$_4$O$_2$)][B(C$_6$F$_5$)$_4$]$_3$·Et$_2$O (6·Et$_2$O). Compound **6** crystallized as red plate crystals. The crystals diffracted poorly, partly because of the plate morphology. After several attempts, the best crystal was chosen for data collection. Good quality data were acquired down to 1.03 Å. As a result, not as many reflections were available for refinement, and thus some restraints were applied to stabilize the refinement. Two half-molecules of $[(NiMe_6tren)_2(C_6H_4O_2)]^{3+}$, along with three $[B(C_6F_5)_4]^-$ anions, were found in the asymmetric unit. The remaining half of the molecule were generated by an inversion symmetry. The second molecule of $[(NiMe_6tren)_2(C_6H_4O_2)]^{3+}$ was found to be disordered by the Me$_6$tren ligand and was modeled over two positions. Refinement of the disordered Me$_6$tren ligand was stabilized by appropriate similar distance restraints. Anisotropic refinement of the disordered Me$_6$tren ligand was stabilized by the enhanced rigid bond restraints. All non-H atoms were refined anisotropically. All H atoms were placed on the geometrically calculated positions using the riding model.

Table S1. Crystal data and structure refinement for **1**–**3**

	$[(FeMe_6tren)_2(C_6H_4O_2)]$-$[B(C_6F_5)_4]_2$ (**1**)	$[[(CoMe_6tren)_2(C_6H_4O_2)]$-$[B(C_6F_5)_4]_2$ (**2**)	$[[(NiMe_6tren)_2(C_6H_4O_2)]$-$[B(C_6F_5)_4]_2$ (**3**)
CCDC Number	1958690	1958691	1958692
Empirical formula	$Fe_2F_{40}O_4N_8C_{86}B_2H_{80}$	$Co_2F_{40}O_4N_8C_{86}B_2H_{80}$	$Ni_2F_{40}O_5N_8C_{90}B_2H_{90}$
Formula weight (g/mol)	2182.90	2189.06	2262.73
Temperature (K)	100(2)	100(2)	100(2)
Wavelength (Å)	0.7728	0.7288	0.7288
Crystal system	Triclinic	Triclinic	Triclinic
Space group	$P\bar{1}$	$P\bar{1}$	$P\bar{1}$
a (Å), α (˚)	12.088(2), 81.857(2)	12.009(3), 82.488(3)	12.015(2), 97.774(3)
b (Å), β (˚)	12.625(2), 89.421(3)	12.574(3), 89.864(3)	12.540(2), 90.679(3)
c (Å), γ (˚)	16.135(3), 77.548(2)	16.141(4), 77.525(3)	16.083(3), 101.898(2)
Volume (Å^3)	2379.7(7)	2358.6(10)	2347.5(7)
Z	1	1	1
Density (calculated, g/cm^3)	1.523	1.541	1.601
Absorption coefficient (mm^{-1})	0.463	0.514	0.572
F(000)	1106	1108	1152
Crystal size (mm^3)	0.143 × 0.130 × 0.071	0.164 × 0.164 × 0.086	0.130 × 0.090 × 0.060
θ range for data collection (°)	1.711 to 30.405	1.716 to 31.877	1.719 to 34.098
Index range	$-16 \le h \le 16$	$-17 \le h \le 17$	$-18 \le h \le 18$
	$-17 \le k \le 17$	$-17 \le k \le 18$	$-19 \le k \le 19$
	$-22 \le l \le 22$	$-23 \le l \le 23$	$-24 \le l \le 24$
Reflections collected	46455	49969	56012
Independent reflections, R_{int}	13359, 0.0635	15029, 0.0697	17900, 0.0514
Completeness to θ = 25.930°	99.9 %	100.0 %	99.9 %
Max. and min. transmission	0.968 and 0.920	0.957 and 0.894	0.9662 and 0.9035
Data / restraints / parameters	13359 / 42 / 656	15029 / 30 / 656	17900 / 25 / 701
Goodness-of-fit on F^2	1.036	1.031	1.049
Final R indices [$I > 2\sigma(I)$]	R_1 = 0.0541, wR_2 = 0.1461	R_1 = 0.0492, wR_2 = 0.1275	R_1 = 0.0393, wR_2 = 0.1013
R indices (all data)	R_1 = 0.0685, wR_2 = 0.1585	R_1 = 0.0622, wR_2 = 0.1380	R_1 = 0.0484, wR_2 = 0.1074
Largest diff. peak and hole (e·Å^{-3})	1.514 and –0.506	0.872 and –0.481	0.603 and –0.722

Table S2. Crystal data and structure refinement for **4**–**6**

	$[[(FeMe_6tren)_2(C_6H_4O_2)]$-$[B(C_6F_5)_4]_3$ (**4**)	$[[(CoMe_6tren)_2(C_6H_4O_2)]$-$[B(C_6F_5)_4]_3$ (**5**)	$[[(NiMe_6tren)_2(C_6H_4O_2)]$-$[B(C_6F_5)_4]_3$ (**6**)
CCDC Number	1958693	1958694	1958695
Empirical formula	$Fe_2F_{60}O_3N_8C_{106}B_3H_{74}$	$Co_2F_{60}O_3N_8C_{106}B_3H_{74}$	$Ni_2F_{60}O_3N_8C_{106}B_3H_{74}$
Formula weight (g/mol)	2791.86	2798.02	2797.58
Temperature (K)	100(2)	100(2)	100(2)
Wavelength (Å)	0.7728	0.7288	0.7288
Crystal system	Monoclinic	Monoclinic	Monoclinic
Space group	$P2_1/c$	$P2_1/c$	$P2_1/c$
a (Å), α (°)	26.788(6), 90	26.750(10), 90	26.694(10), 90
b (Å), β (°)	22.494(5), 110.506(3)	22.475(8), 110.625(4)	22.411(8), 110.424(4)
c (Å), γ (°)	19.436(5), 90	19.437(7), 90	19..450(7), 90
Volume ($Å^3$)	10970(4)	10936(7)	10905(7)
Z	4	4	4
Density (calculated, g/cm^3)	1.690	1.699	1.704
Absorption coefficient (mm^{-1})	0.449	0.491	0.537
F(000)	5588	5596	5604
Crystal size (mm^3)	0.110 × 0.086 × 0.036	0.190 × 0.140 × 0.057	0.050 × 0.030 × 0.015
θ range for data collection (°)	1.247 to 24.462	1.249 to 23.068	1.251 to 20.718
Index range	$-30 \le h \le 30$	$-28 \le h \le 28$	$-25 \le h \le 25$
	$-25 \le k \le 25$	$-24 \le k \le 24$	$-21 \le k \le 21$
	$-22 \le l \le 22$	$-20 \le l \le 20$	$-18 \le l \le 18$
Reflections collected	146345	119921	91959
Independent reflections, R_{int}	16841, 0.0703	14215, 0.0784	10441, 0.0905
Completeness to θ, θ (°)	99.9 %, 24.462	99.9 %, 23.068	100.0 %, 20.718
Max. and min. transmission	0.9583 and 0.807	0.9579 and 0.792	0.992 and 0.721
Data / restraints / parameters	16841 / 198 / 1795	14215 / 199 / 1804	10441 / 113 / 1712
Goodness-of-fit on F^2	1.041	1.136	1.028
Final R indices [$I > 2\sigma(I)$]	$R_1 = 0.0445$, $wR_2 = 0.1057$	$R_1 = 0.0617$, $wR_2 = 0.1440$	$R_1 = 0.0487$, $wR_2 = 0.1155$
R indices (all data)	$R_1 = 0.0633$, $wR_2 = 0.1159$	$R_1 = 0.0783$, $wR_2 = 0.1528$	$R_1 = 0.0651$, $wR_2 = 0.1271$
Largest diff. peak and hole ($e \cdot Å^{-3}$)	0.731 and –0.554	1.177 and –0.697	1.048 and –0.503

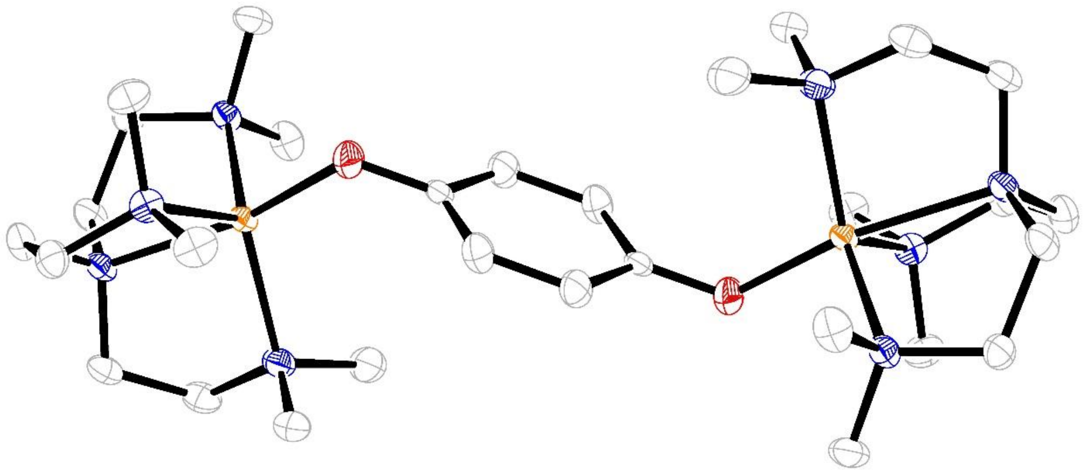

Figure S1. Solid-state structure of $[(Me_6tren)_2Fe_2(C_6H_4O_2)]^{2+}$, as observed in **1**·2THF·Et_2O, with thermal ellipsoids shown at the 50% probability level. Orange, red, blue, and gray ellipsoids represent Fe, O, N, and C atoms, respectively; H atoms are omitted for clarity. Data were collected at 100 K.

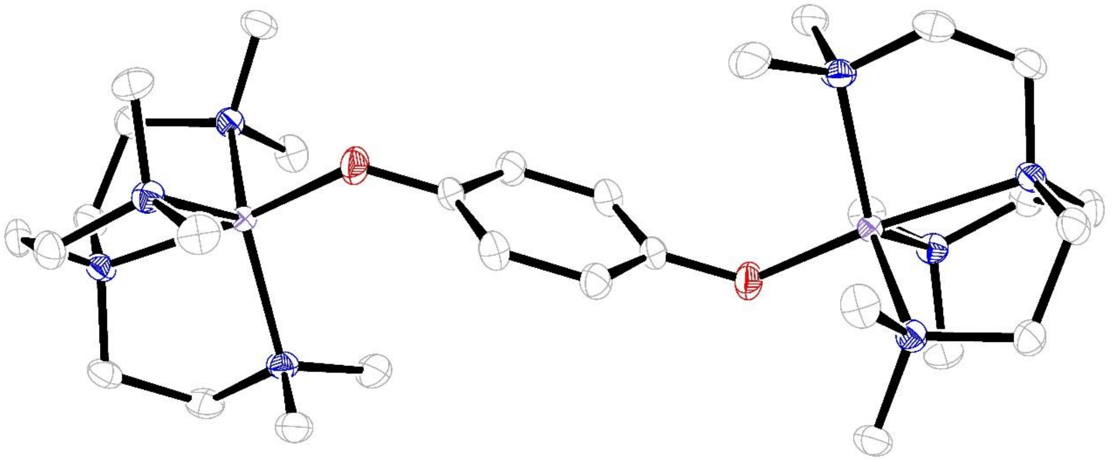

Figure S2. Solid-state structure of $[(Me_6tren)_2Co_2(C_6H_4O_2)]^{2+}$, as observed in **2**·2THF·Et_2O, with thermal ellipsoids at the 50% probability level. Purple, red, blue, and gray ellipsoids represent Co, O, N, and C atoms, respectively; H atoms are omitted for clarity. Data were collected at 100 K.

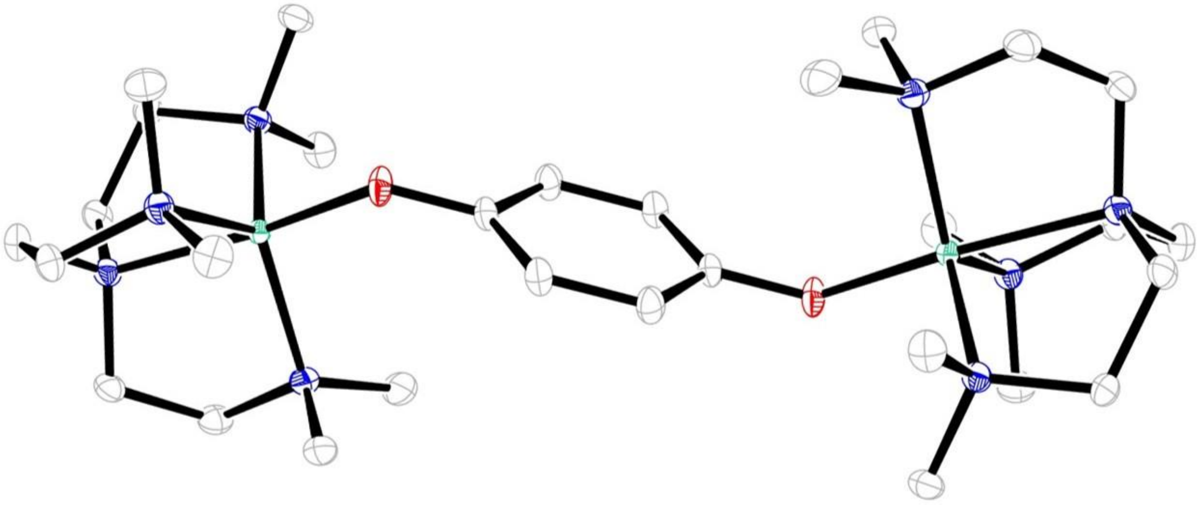

Figure S3. Solid-state structure of $[(Me_6tren)_2Ni_2(C_6H_4O_2)]^{2+}$, as observed in **3**·2THF·Et_2O, with thermal ellipsoids at the 50% probability level. Green, red, blue, and gray ellipsoids represent Ni, O, N, and C atoms, respectively; H atoms are omitted for clarity. Data were collected at 100 K.

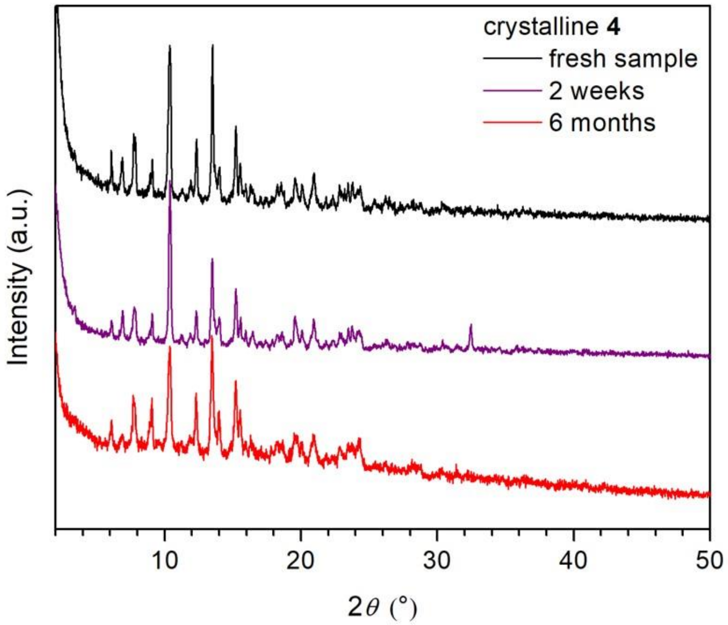

Figure S4. Powder X-ray diffraction patterns of crystalline **4**·Et_2O following air exposure for 1 min (black), 2 weeks (purple), and 6 months (red). Small amount of amorphous decomposition product begins to develop after 6 months, as seen in the growing slanted background of the red pattern.

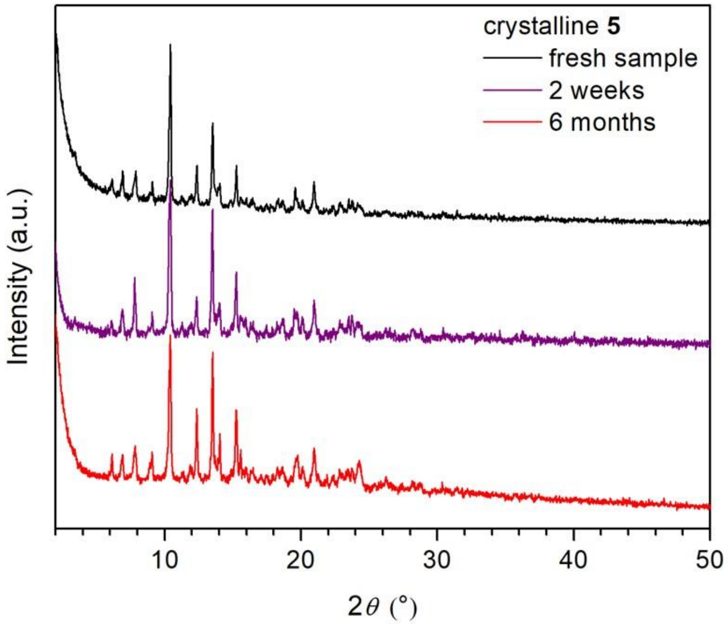

Figure S5. Powder X-ray diffraction patterns of crystalline **5**·Et_2O upon exposure to air for 1 min (black), 2 weeks (purple), and 6 months (red). The sample remains crystalline over 6 months.

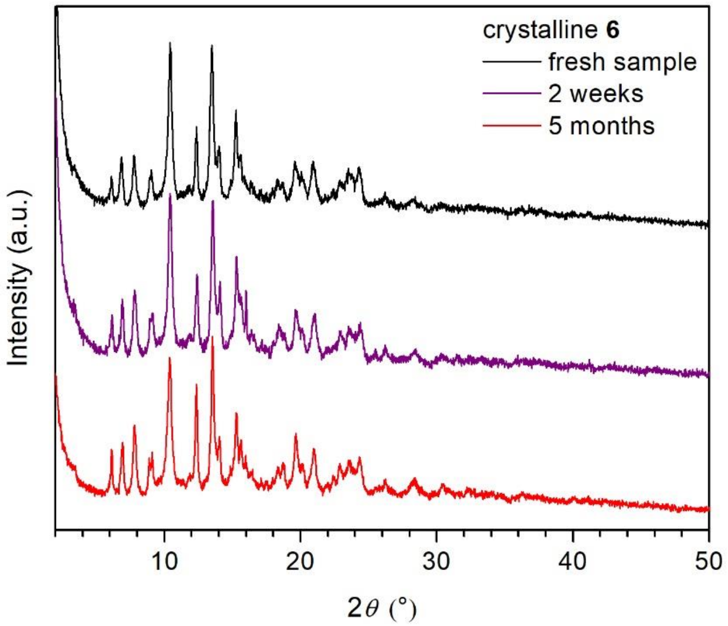

Figure S6. Powder X-ray diffraction patterns of crystalline **6**·Et_2O upon exposure to air for 1 min (black), 2 weeks (purple), and 5 months (red). The sample remains crystalline over 5 months.

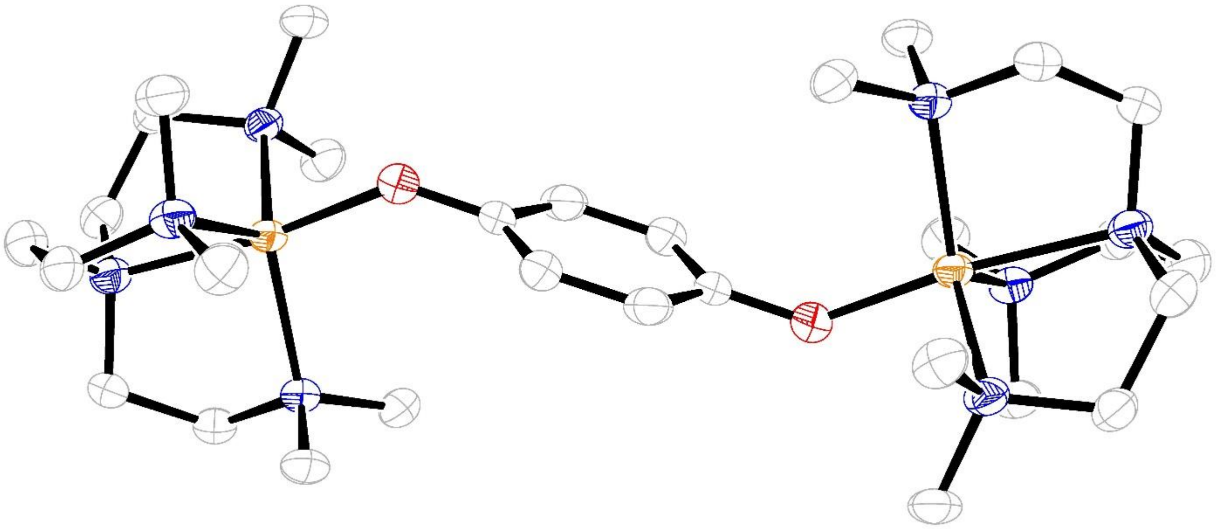

Figure S7. Solid-state structure of $[(Me_6tren)_2Fe_2(C_6H_4O_2)]^{3+}$, as observed in **4**·$Et_2O$, with thermal ellipsoids shown at the 50% probability level. Orange, red, blue, and gray ellipsoids represent Fe, O, N, and C atoms, respectively; H atoms are omitted for clarity. Data were collected at 100 K.

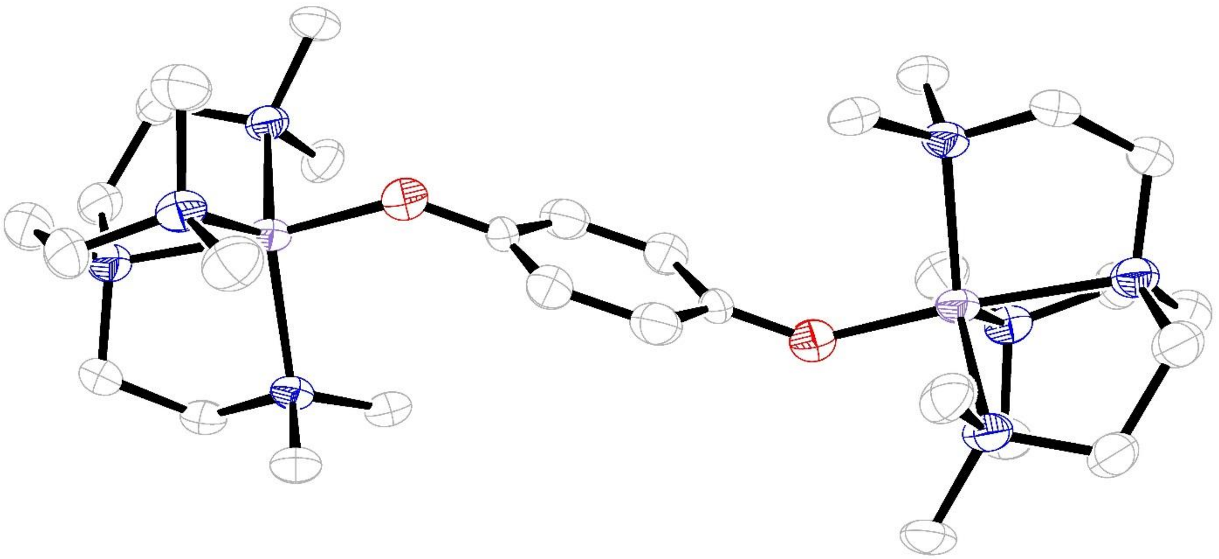

Figure S8. Solid-state structure of $[(Me_6tren)_2Co_2(C_6H_4O_2)]^{3+}$, as observed in **5**·$Et_2O$, with thermal ellipsoids at the 50% probability level. Purple, red, blue, and gray ellipsoids represent Co, O, N, and C atoms, respectively; H atoms are omitted for clarity. Data were collected at 100 K.

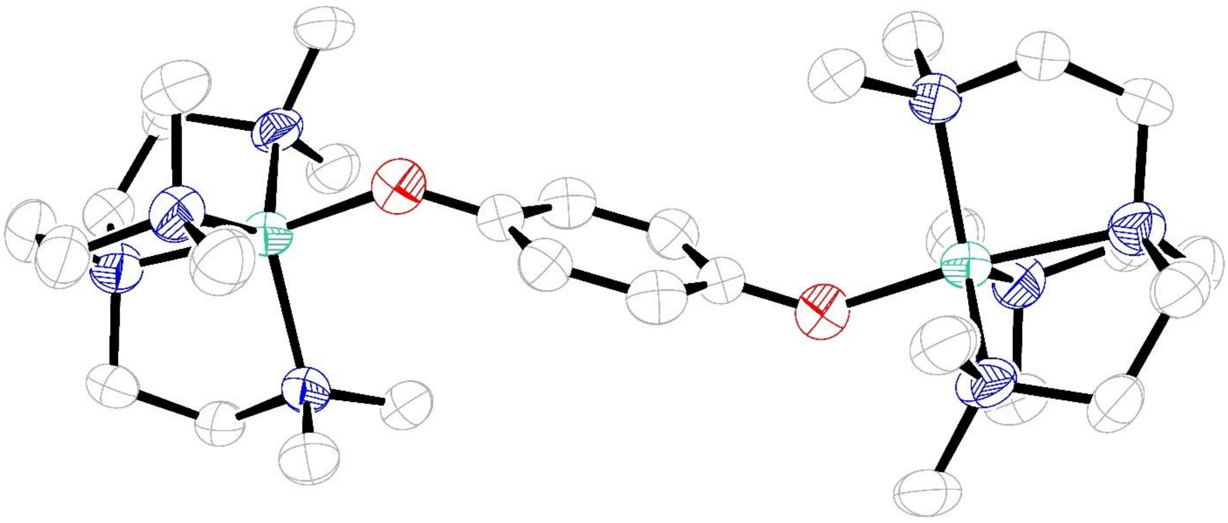

Figure S9. Solid-state structure of $[(Me_6tren)_2Ni_2(C_6H_4O_2)]^{3+}$, as observed in **6**·$Et_2O$, with thermal ellipsoids at the 50% probability level. Green, red, blue, and gray ellipsoids represent Ni, O, N, and C atoms, respectively; H atoms are omitted for clarity. Data were collected at 100 K.

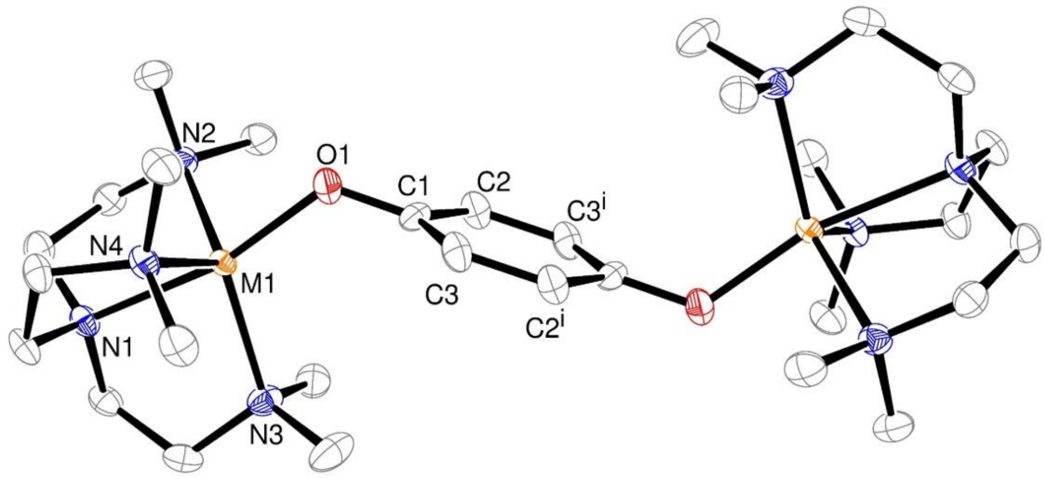

Numbering scheme for $[(Me_6tren)_2M_2(C_6H_4O_2)]^{n+}$ (M = Fe, Co, Ni; n = 2, 3).

Table S3. Selected bond distances (Å) and angles (°) for **1**–**3**.

	**1** (Fe)	**2** (Co)	**3** (Ni)
M1–O1	1.9019(17)	1.8839(14)	1.8879(9)
M1–N1	2.2653(19)	2.2207(15)	2.1207(10)
M1–N2	2.227(2)	2.1426(16)	2.1009(11)
M1–N3	2.211(2)	2.1717(15)	2.1215(11)
M1–N4	2.1911(19)	2.1607(16)	2.2348(10)
O1–M1–N1	167.10(7)	169.34(6)	166.39(4)
O1–M1–N2	89.94(8)	101.85(6)	102.19(4)
O1–M1–N3	112.41(8)	90.00(6)	104.04(4)
O1–M1–N4	98.84(7)	106.23(6)	85.13(4)
N2–M1–N3	117.99(7)	119.11(6)	111.26(4)
N2–M1–N4	118.29(7)	115.45(6)	121.40(4)
N3–M1–N4	114.22(8)	117.71(6)	123.19(4)
Σ[N(eq)–M–N(eq)]	350.50	352.27	355.85
N1–M1–N2	79.10(7)	80.95(6)	84.26(4)
N1–M1–N3	79.10(7)	79.74(6)	84.33(4)
N1–M1–N4	80.71(7)	81.37(6)	81.29(4)
O1–C1	1.319(3)	1.343(2)	1.3405(14)
C1–C2	1.444(3)	1.393(3)	1.4024(17)
C1–C2	1.382(3)	1.399(3)	1.4032(16)
C2–C3^{i}	1.398(3)	1.390(3)	1.3914(17)
C1–O1–M1	124.91(15)	131.33(12)	132.08(8)
C–C (avg)	1.408	1.394	1.399

Table S4. Selected bond distances (Å) and angles (°) for **4**–**6**. Values in each case are for the first molecule in the crystal structure, as described in the main text.

	**4** (Fe)	**5** (Co)	**6** (Ni)
M1–O1	1.936(3)	1.922(5)	1.928(4)
M1–N1	2.202(3)	2.176(5)	2.070(5)
M1–N2	2.177(3)	2.138(5)	2.078(5)
M1–N3	2.182(3)	2.158(6)	2.135(5)
M1–N4	2.178(3)	2.131(5)	2.161(5)
O1–M1–N1	171.09(11)	171.4(2)	169.6(2)
O1–M1–N2	106.51(11)	100.2(2)	101.75(18)
O1–M1–N3	91.13(11)	90.6(2)	97.98(19)
O1–M1–N4	97.64(11)	103.37(19)	87.7(2)
N2–M1–N3	115.99(11)	122.19(19)	113.16(19)
N2–M1–N4	114.18(11)	115.7(2)	114.78(19)
N3–M1–N4	123.80(11)	116.4(2)	129.3(18)
Σ[N(eq)–M–N(eq)]	353.97	354.29	357.24
N1–M1–N2	81.48(11)	82.0(2)	86.11(19)
N1–M1–N3	81.65(11)	81.2(2)	84.92(19)
N1–M1–N4	82.24(11)	82.89(19)	82.8(2)
O1–C1	1.291(4)	1.288(7)	1.296(7)
C1–C2	1.416(5)	1.436(10)	1.387(9)
C1–C2	1.413(5)	1.386(10)	1.419(9)
C2–C3^{i}	1.363(5)	1.362(10)	1.375(9)
C1–O1–M1	135.1(2)	138.1(4)	136.6(4)
C–C (avg)	1.397	1.395	1.394

Density Functional Theory Calculations

To compute the strength of magnetic exchange in **6**, DFT calculation was performed on the $[(NiMe_6tren)_2(C_6H_4O_2)]^{3+}$ cation in the structure of **6** with B3LYP functional and 6-31+G(d) basis set. Atomic coordinates were taken from the crystal structure of the first (non-disordered) molecule. Broken symmetry calculation was performed on the sextet $S = {}^5/_2$ state and the broken-symmetry $S = {}^3/_2$ state. Magnetic exchange constant was calculated from the energies of the high spin sextet (E_{HS}) and broken-symmetry quartet (E_{BS}) states:[9,10]

$$J = \frac{E_{BS} - E_{HS}}{\langle S^2 \rangle_{HS} - \langle S^2 \rangle_{BS}}$$

	High spin (S = 5/2)	Broken-symmetry (S = 3/2)
E (Hartree)	–4784.9614	–4784.9718
$\langle S^2 \rangle$	8.76516	4.55393

Computation results indicates that the $S = {}^3/_2$ state is the ground state, and the high spin $S = {}^5/_2$ state lies 2284 cm^{-1} above the ground state, which gives J = –542 cm^{-1}. The results confirm that compound **6** can be regarded as a giant spin $S = {}^3/_2$ system that is persistent up to 300 K.

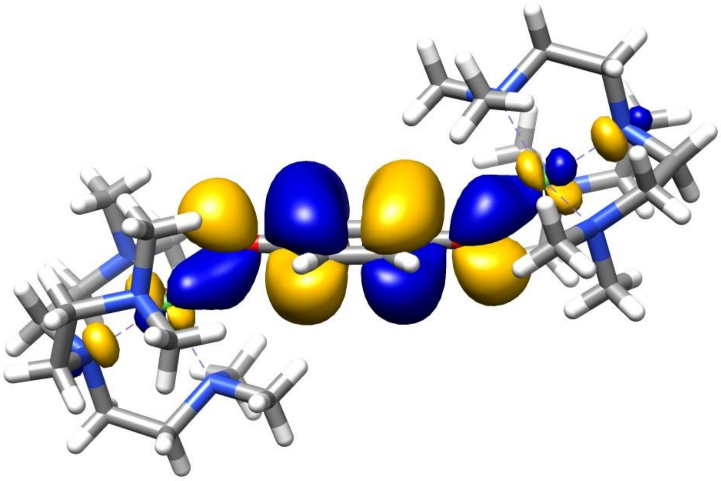

Figure S10. Molecular orbital representation showing the highest (singly) occupied beta molecular orbital in **6** centered on the semiquinone radical, plotted with contour surface of electron density ±0.020. Small direct overlap with metal-based d orbitals was observed. This representation shows that the unpaired electron resides in the expected π* orbital of the semiquinone. Direct orbital overlap with the two Ni $3d_{xz}$ orbitals is clearly visible in the plot. Natural Bond Orbital analysis indicates an occupancy of 1.85 electrons on the d_{xz} orbital.

Vibrational frequencies were calculated using unrestricted DFT/B3LYP and 6-31+G(d) basis set on the $[(NiMe_6tren)_2(C_6H_4O_2)]^{2+}$ and $[(NiMe_6tren)_2(C_6H_4O_2)]^{3+}$ cations. The structures were taken from the crystal structure of **3** and **6**, respectively, and were optimized prior to frequency calculation.

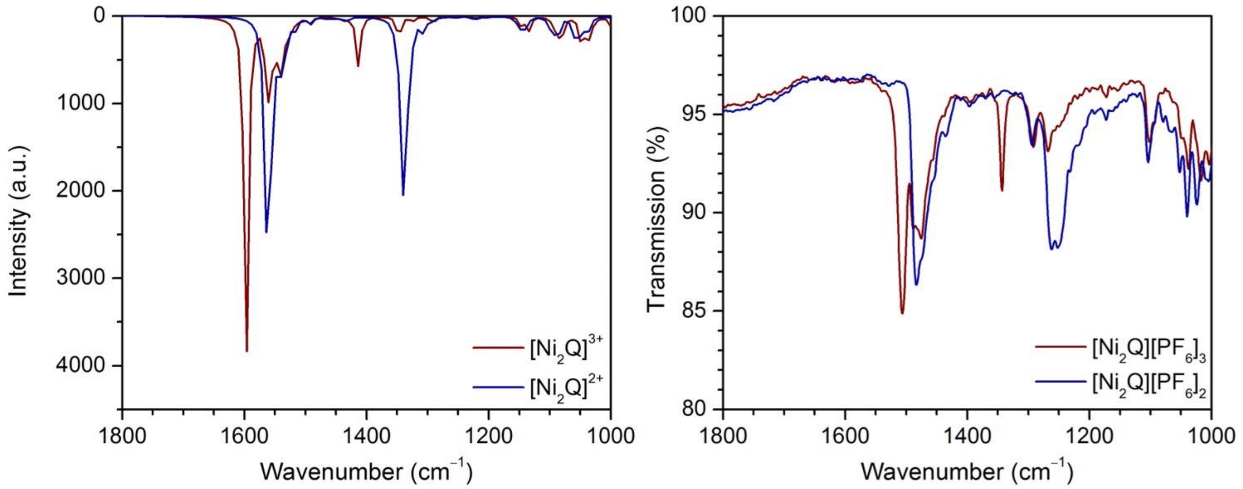

Figure S11. Calculated vibrational spectra (left) and experimental FTIR (right) of the $[(NiMe_6tren)_2(C_6H_4O_2)]^{2+}$ (blue lines) and $[(NiMe_6tren)_2(C_6H_4O_2)]^{3+}$ (red lines).

The calculation predicts two intense absorptions in the IR region, corresponding to the asymmetric C–O stretching of the hydroquinone and semiquinone, and C–H rocking. These vibrations shift to higher energy upon oxidation from hydroquinone to semiquinone, consistent with the experimental FTIR spectra of $[(NiMe_6tren)_2(C_6H_4O_2)](PF_6)_2$ and $[(NiMe_6tren)_2(C_6H_4O_2)](PF_6)_3$ (see the main text).

Spectroscopy

Infrared spectra were collected on a PerkinElmer Avatar Spectrum 400 FTIR spectrophotometer equipped with a Pike attenuated total reflectance accessory (ATR).

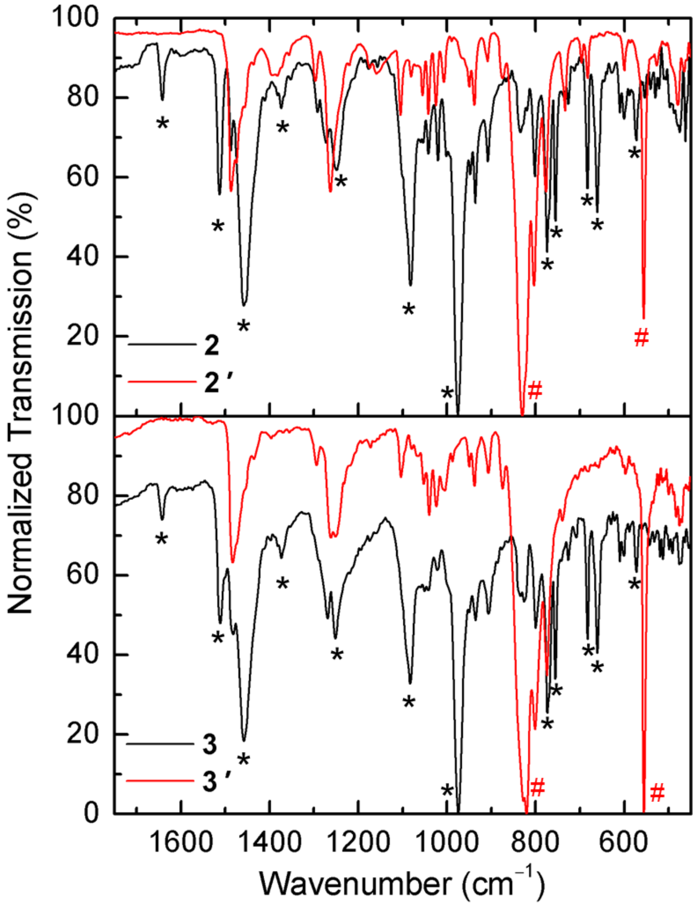

Figure S12. FTIR spectra of **2** and **2′** (top), and **3** and **3′** (bottom). Black asterisks denote vibrational modes of the $[B(C_6F_5)_4]^-$ anion, and red pound signs denote vibrational modes of the PF_6^- anion.

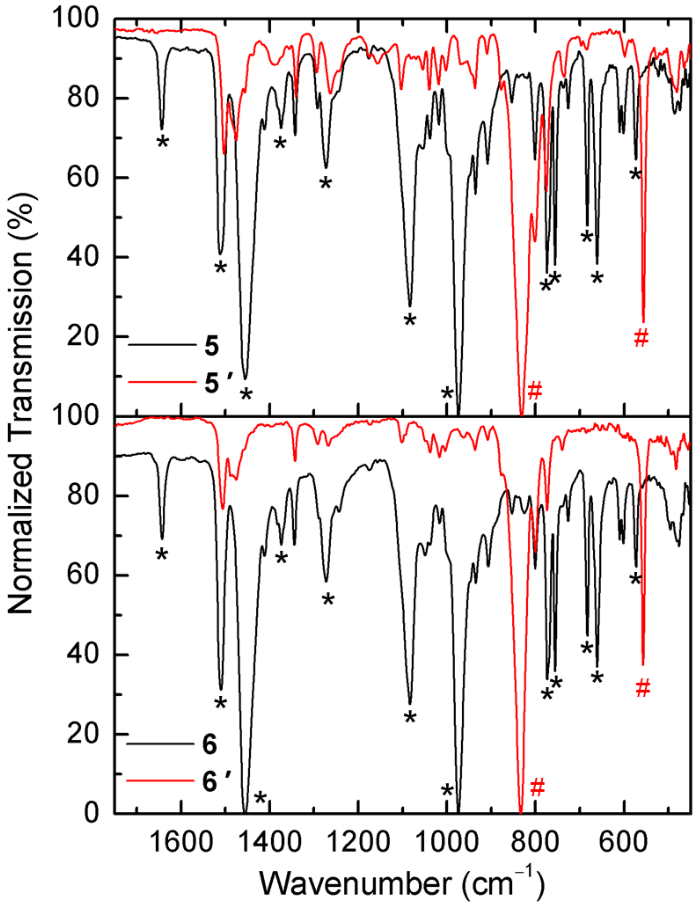

Figure S13. FTIR spectra of **5** and **5′** (top), and **6** and **6′** (bottom). Black asterisks denote vibrational modes of the $[B(C_6F_5)_4]^-$ anion, and red pound signs denote vibrational modes of the PF_6^- anion.

Zero-field ^{57}Fe Mössbauer spectra were recorded in a constant acceleration SEE Co. spectrometer (Minneapolis, MN) which utilized a cobalt-57 source embedded in a Rh matrix. Spectra were collected between room temperature and 5 K in a Janis Research Co. cryostat (Willmington, MA). Collected spectra were analyzed using the WMOSS software package (SEE Co. Minneapolis, MN). Isomer shifts are reported relative to α-iron (30 μm thickness) at 295 K. Samples were placed in nylon washers wrapped in Kapton® tape. The spectrum of **1** features a Lorentzian doublet with a narrow linewidth of 0.253(2) mm/s at 5 K (Figure S14, top), consistent with one iron environment. The isomer shift of 1.0320(6) mm/s and a large quadrupole splitting of 3.198(1) mm/s is consistent with a high-spin iron(II) center. The isomer shift is slightly lower than typical Fe^{2+} centers in octahedral environments, indicating that the iron center is in a lower coordination number, as expected for a five-coordinate geometry.

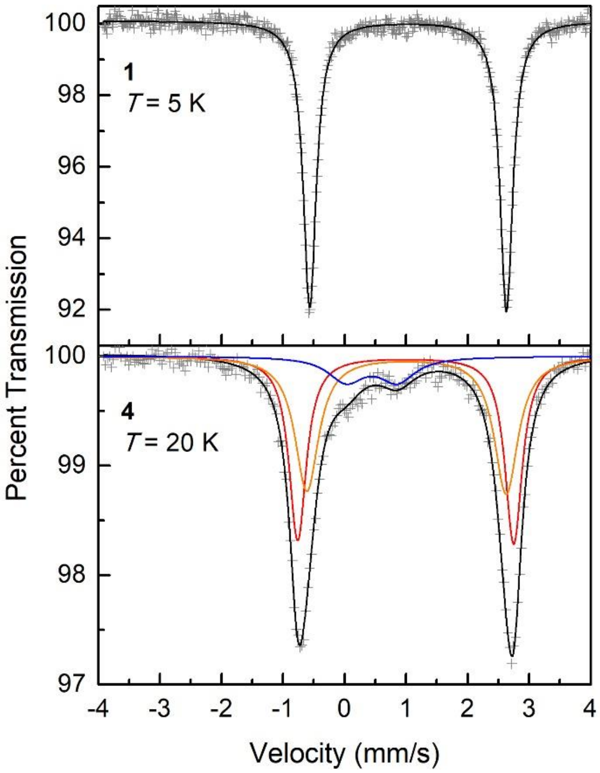

Figure S14. ^{57}Fe Mössbauer spectra of **1** (top) obtained at 5 K and **4** (bottom) collected at 20 K (grey crosses). Black lines represent the total fit to each spectrum. For the spectrum of **4**, orange and red lines represent the fit to the two distinct molecule in the crystal structure, and blue line represents a high spin iron(III) impurity.

Table S5. ^{57}Fe Mössbauer fitting parameters for $[(FeMe_6tren)_2(C_6H_4O_2)][B(C_6F_5)_4]_2$ (**1**)

Temperature	5 K	80 K
δ (mm/s)	1.0320(6)	1.0150(8)
ΔE_Q (mm/s)	3.198(1)	3.024(2)
Γ (mm/s)	0.253(2)	0.256(3)
χ^2	0.666	0.718

The Mössbauer spectra of **4** collected at 20 K features a broad quadrupole doublet. In addition, a shoulder was observed around 0.8 mm/s. Variable-temperature Mössbauer measurement has revealed the nature of the sample as consisting of two distinct high spin Fe(II) sites, possibly corresponding to the two distinct molecules of $[(FeMe_6tren)_2(C_6H_4O_2)]^{3+}$ in the crystal structure which features different bond metrics (Figure S15). The relative area of these two species were constrained to be the same as imposed by the crystal structure. In addition, a high spin Fe(III) impurity was observed in the sample (blue line in the fit). Analysis of the temperature dependence of the absorbance area suggests that this species behaves independently from the high spin Fe(II), with the Debye temperature of 175 K. On the other hand, the Debye temperature for the high spin

Fe(II) species is 110 K in **4** (Figure S16). The Debye temperatures for the two species are consistent with typical organometallic iron complexes.[11,12]

Table S6. ^{57}Fe Mössbauer fitting parameters for $[(FeMe_6tren)_2(C_6H_4O_2)][B(C_6F_5)_4]_3$ (**4**)

Temperature	20 K	50 K	80 K	295 K
δ_1 (mm/s)	0.996(2)	0.989(2)	0.981(2)	0.902(18)
ΔE_{Q1} (mm/s)	3.528(8)	3.430(5)	3.343(4)	2.23(3)
Γ_1 (mm/s)	0.313(7)	0.258(6)	0.266(5)	0.28(4)
Relative Area	0.449(5)	0.439(4)	0.427(5)	0.28(3)
δ_2 (mm/s)	1.008(4)	1.011(5)	1.002(4)	0.906(23)
ΔE_{Q2} (mm/s)	3.23(1)	3.07(1)	2.90(1)	1.67(4)
Γ_2 (mm/s)	0.42(2)	0.44(1)	0.48(1)	0.33(5)
Relative Area	0.449(5)	0.439(4)	0.427(5)	0.28(3)
δ_3 (mm/s)	0.42(4)	0.45(1)	0.43(2)	0.25(4)
ΔE_{Q3} (mm/s)	0.82(6)	0.83(3)	0.78(3)	0.52(13)
Γ_3 (mm/s)	0.62(8)	0.41(4)	0.54(5)	0.72(16)
Relative Area	0.106(8)	0.121(8)	0.147(8)	0.43(5)
χ^2	0.625	0.760	0.842	0.841

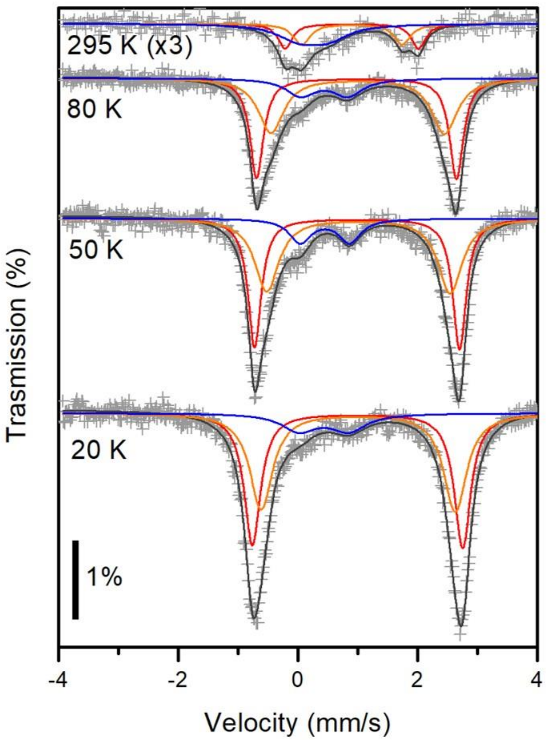

Figure S15. Variable-temperature Mössbauer spectra of **4** (grey crosses). The black bar at the bottom left represents the height of a 1% transmission. The spectrum at 295 K was magnified by

three times to improve clarity. Black lines represent total fits to the data. Red, orange, and blue lines represent the two high spin Fe(II) sites and the high spin Fe(III) impurity (see Table S6).

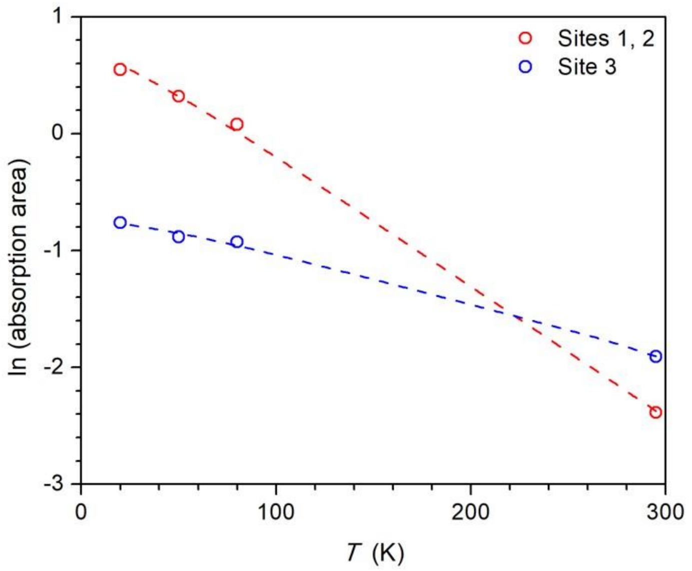

Figure S16. Temperature dependence of the spectral absorption area of **4**. Red circles represent one of the two high spin Fe(II) sites (the area of the two sites were constrained to be the same), and blue circles represent the high spin Fe(III) impurity. Dashed lines represent fit to the data with the Debye model for a solid.[13]

UV-vis-NIR spectra were collected on a Cary 5000 spectrophotometer in the range 200–2500 nm on THF solutions of **1**–**6**.

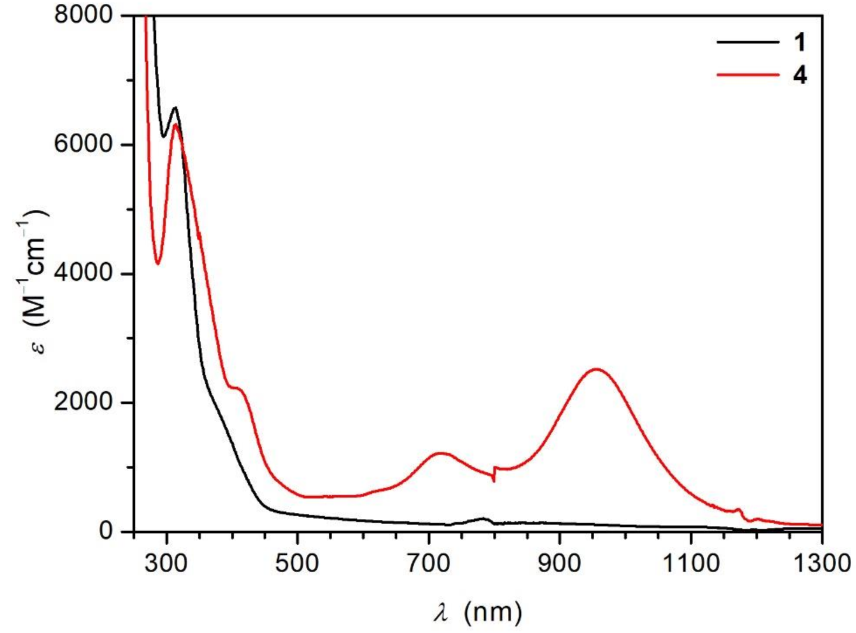

Figure S17. UV-vis-NIR spectra of $[(FeMe_6tren)_2(C_6H_4O_2)][B(C_6F_5)_4]_2$ (**1**) and $[(FeMe_6tren)_2(C_6H_4O_2)][B(C_6F_5)_4]_3$ (**4**) in THF solution.

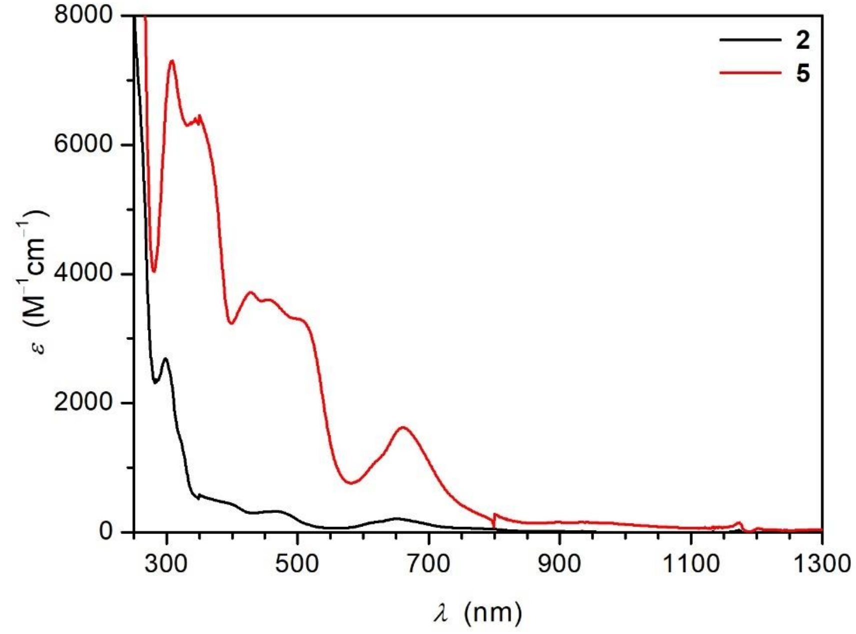

Figure S18. UV-vis-NIR spectra of $[(CoMe_6tren)_2(C_6H_4O_2)][B(C_6F_5)_4]_2$ (**2**) and $[(CoMe_6tren)_2(C_6H_4O_2)][B(C_6F_5)_4]_3$ (**5**) in THF solution.

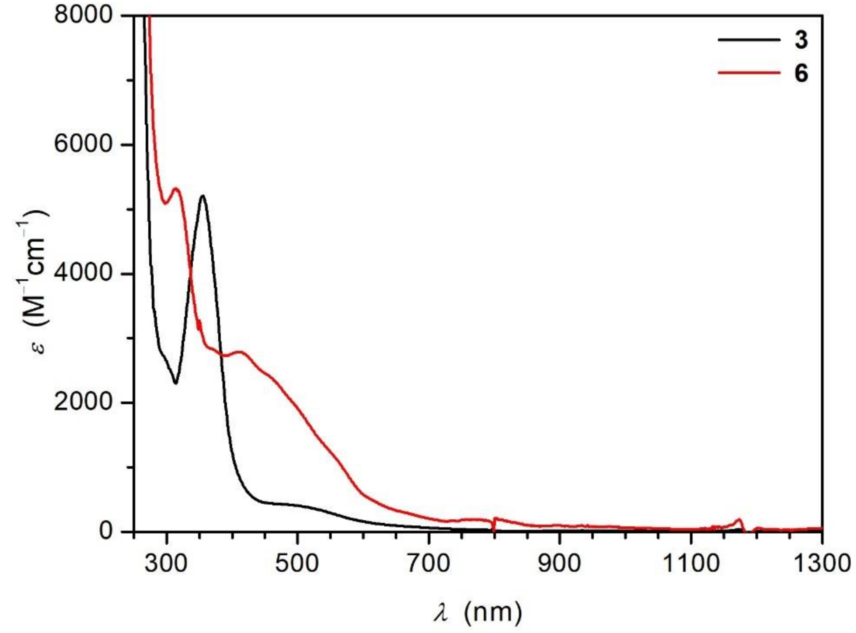

Figure S19. UV-vis-NIR spectra of $[(NiMe_6tren)_2(C_6H_4O_2)][B(C_6F_5)_4]_2$ (**3**) and $[(NiMe_6tren)_2(C_6H_4O_2)][B(C_6F_5)_4]_3$ (**6**) in THF solution.

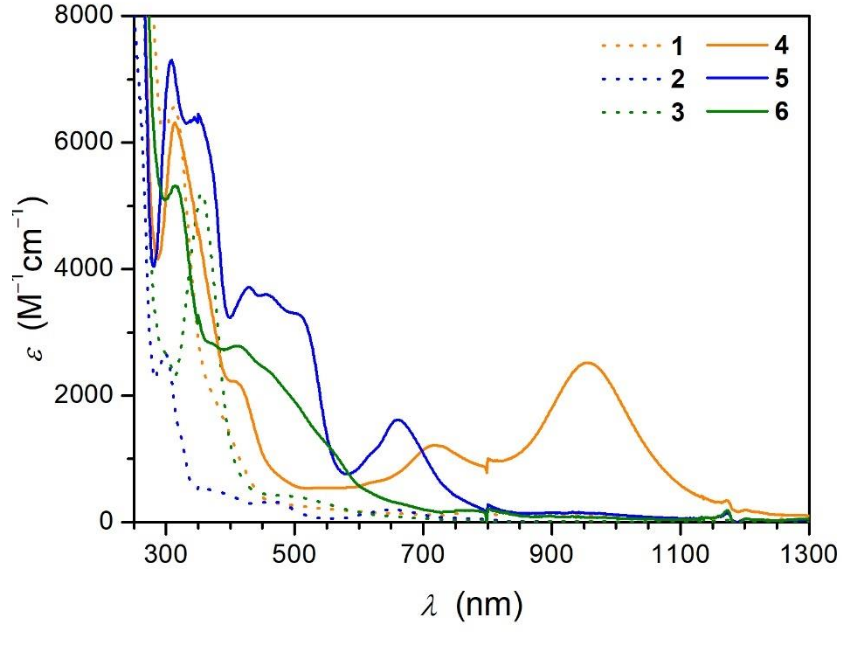

Figure S20. Combined UV-Vis-NIR spectra of the semiquinone complexes **4**–**6** (solid lines), plotted against the hydroquinone complexes **1**–**3** (dotted lines), highlighting the appearance of several transitions in the visible-NIR region.

Magnetic Measurements

Magnetic measurements were performed using a Quantum Design MPMS-XL SQUID magnetometer on polycrystalline samples restrained in eicosane for **1**·2THF·Et_2O, **2**·2THF·Et_2O, **3**·2THF·Et_2O, **4**·Et_2O, **5**·Et_2O, and **6**·Et_2O. Samples were loaded into quartz tubes with a known amount of eicosane, flame sealed under static vacuum, and then heated gently to melt the eicosane and restrain the samples. All data were corrected for diamagnetic contributions from eicosane (χ_d = –2.4306×10^{-4} cm^3/mol) and from the compounds, estimated using Pascal's constants[14] (χ_d = −1.1907×10^{-4} cm^3/mol for **1**·2THF·Et_2O, −1.1887×10^{-3} cm^3/mol for **2**·2THF·Et_2O, −1.887×10^{-3} cm^3/mol for **3**·2THF·Et_2O, −1.3731×10^{-3} cm^3/mol for **4**·Et_2O, −1.3711×10^{-3} cm^3/mol for **5**·Et_2O, −1.3711×10^{-3} cm^3/mol for **6**·Et_2O). Dc magnetic susceptibility data were collected under a field of 1 T. Variable-temperature, variable-field magnetization data were collected under applied fields from 1 to 7 T over the temperature range 2 to 20 K. Magnetization data and dc susceptibility data for **1**–**3** were fit simultaneously using the Hamiltonian in eq 1 in the manuscript, and magnetization and susceptibility data for **4** and **5** were fit simultaneously to eq 2 in the manuscript. The magnetization data for **4–6** at low temperature satisfy the giant spin approximation, and were fitted to the following spin Hamiltonian to obtain molecular zero-field splitting parameters:

$$\hat{H} = D_{mol}\hat{S}_z^2 + E_{mol}\left(\hat{S}_x^2 - \hat{S}_y^2\right) + \mu_B g S \cdot H \quad \text{(S1)}$$

where D_{mol} and E_{mol} are the axial and transverse zero-field splitting parameters of the molecule, S is the spin of the complex in the giant spin approximation (S = $^7/_2$ for **4**, $^5/_2$ for **5**, and $^3/_2$ for **6**), $S_{x/y/z}$ are projections of S onto the x, y, or z axis, respectively, μ_B is the Bohr magneton, g is the Landé g-factor, and H is the magnetic field. All dc susceptibility and magnetization data were fit using the program PHI.[15]

Ac magnetic susceptibility data were collected using a 4 Oe oscillating ac field and applied fields where indicated, and were fit using a generalized Debye model.[16] Standard deviations of the ac susceptibility parameters were obtained using a SolverAid macro operated in tandem with the Solver function in Excel.[17]

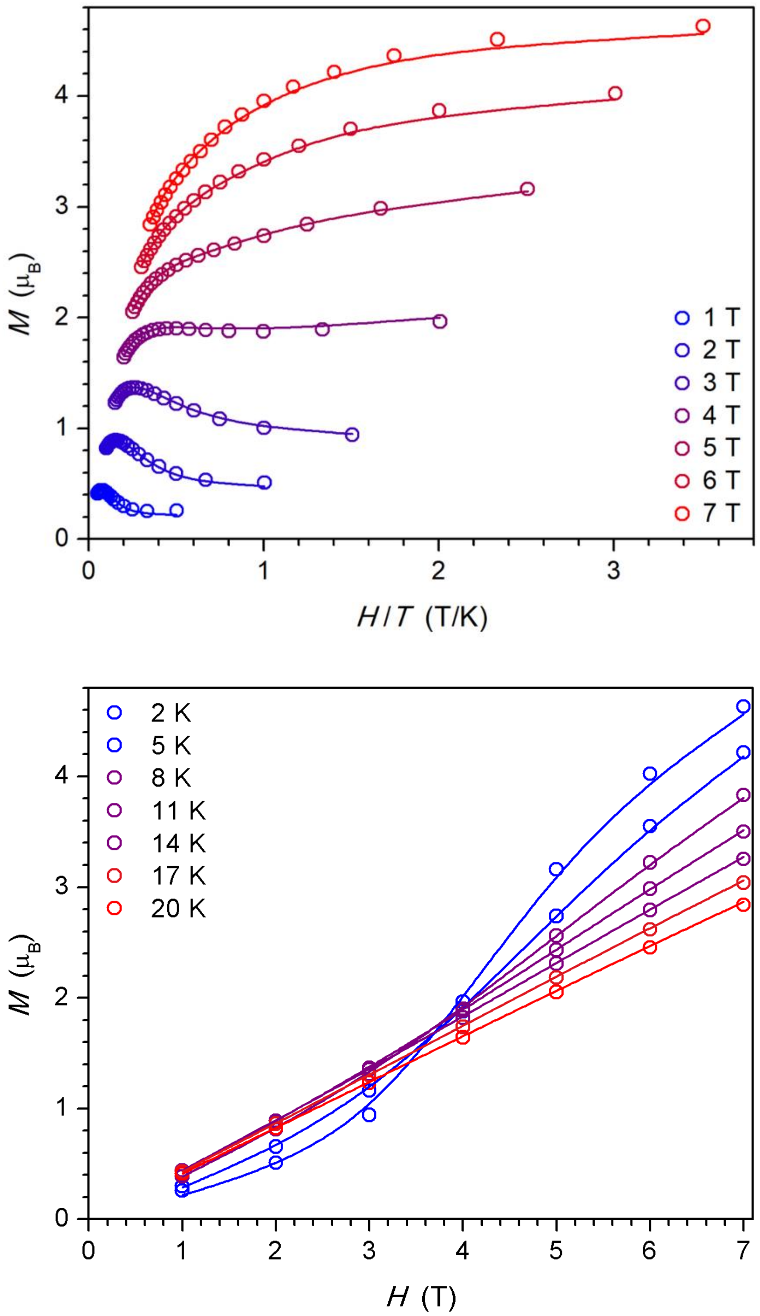

Figure S21. Reduced magnetization (M vs H/T) plot (top) and magnetization (M vs H) plot (bottom) of **1** collected under fields of 1 to 7 T from 2 to 20 K in 1 T and 1 K intervals. Solid lines are fits to the data according to eq 1. Some data were omitted from the magnetization plot for clarity.

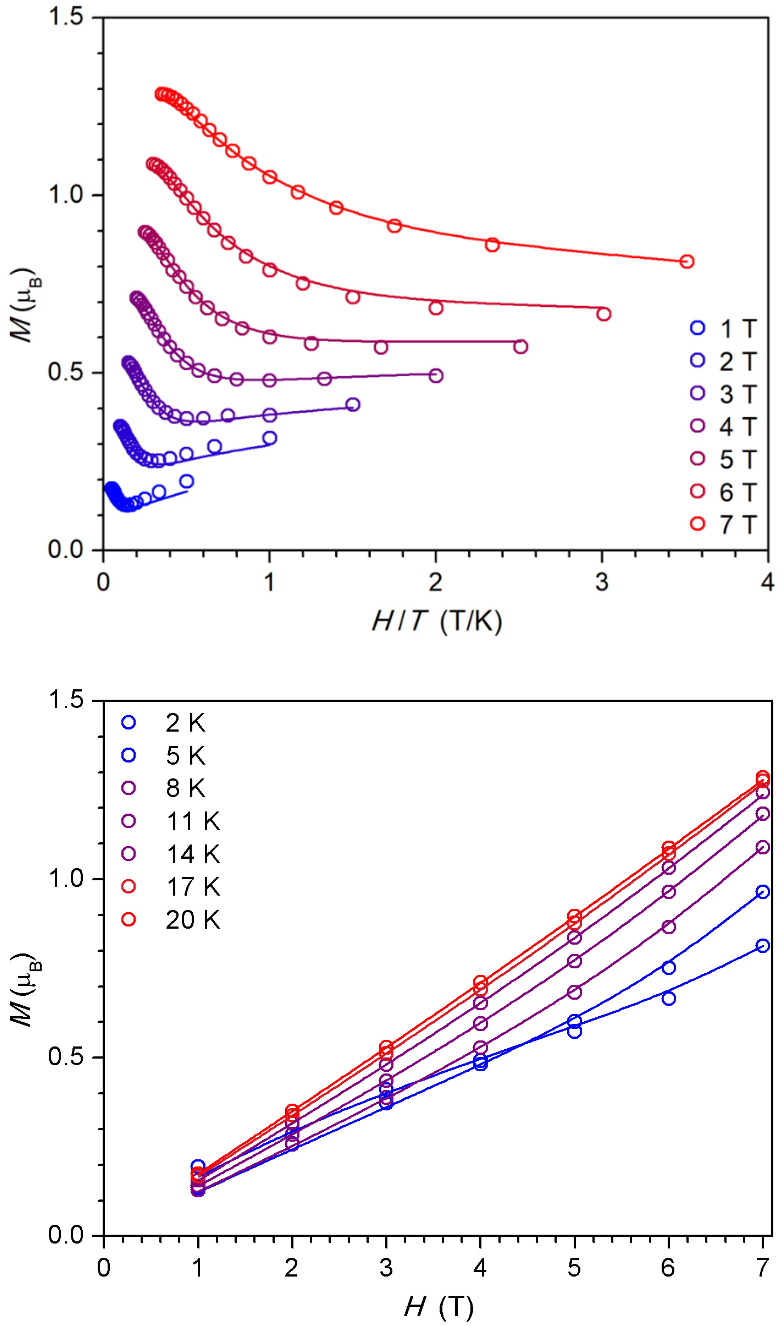

Figure S22. Reduced magnetization (M vs H/T) plot (top) and magnetization (M vs H) plot (bottom) of **2** collected under fields of 1 to 7 T from 2 to 20 K in 1 T and 1 K intervals. Solid lines are fits to the data according to eq 1. Additionally, an $S = {}^3/_2$ impurity was included in the fit, with a fitted contribution of 5.4(1)%. Some data were omitted from the magnetization plot for clarity.

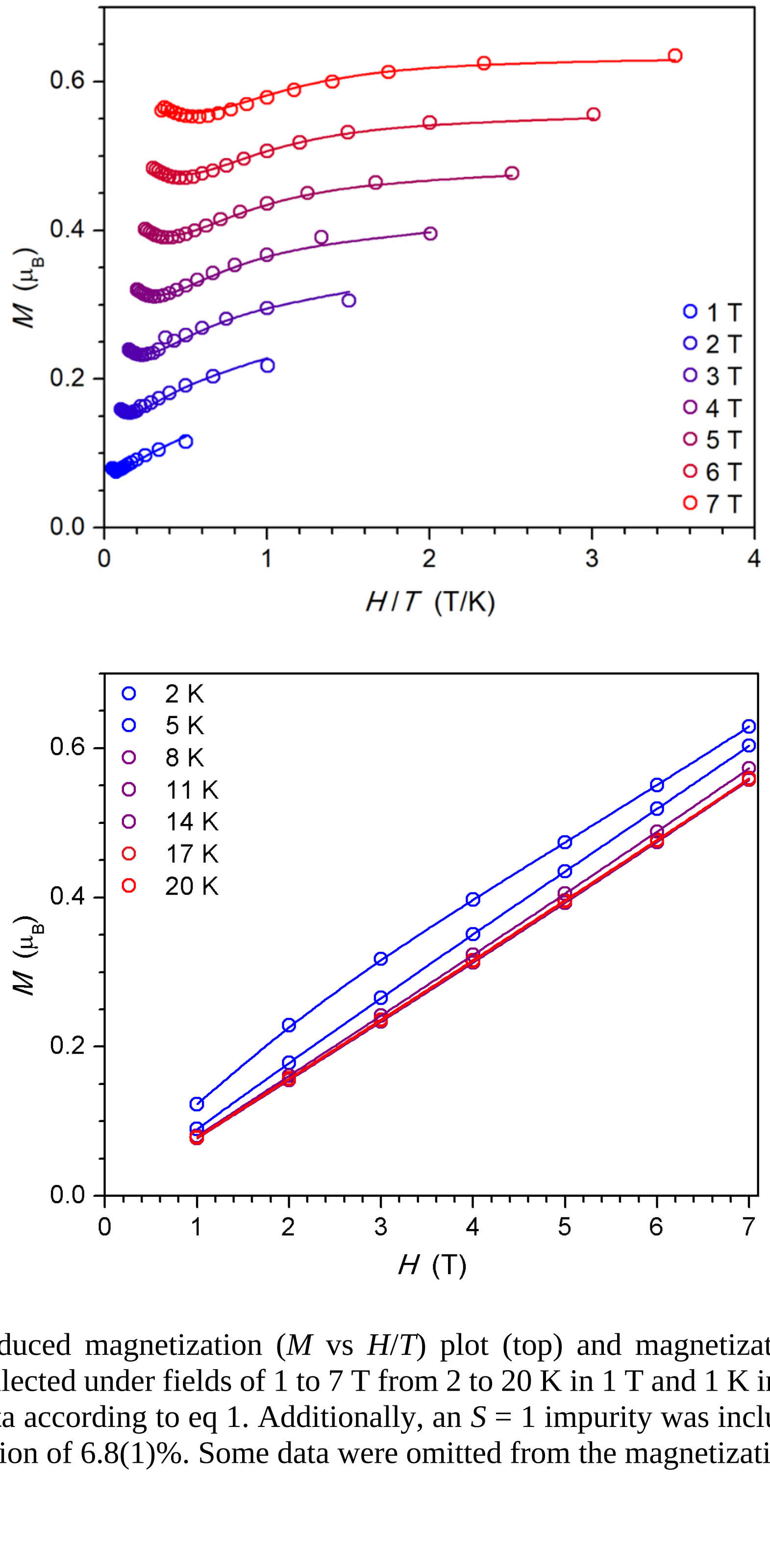

Figure S23. Reduced magnetization (M vs H/T) plot (top) and magnetization (M vs H) plot (bottom) of **3** collected under fields of 1 to 7 T from 2 to 20 K in 1 T and 1 K intervals. Solid lines are fits to the data according to eq 1. Additionally, an S = 1 impurity was included in the fit, with a fitted contribution of 6.8(1)%. Some data were omitted from the magnetization plot for clarity.

Table S7. Fitting parameters for dc susceptibility and magnetization measurements of the hydroquinone-bridged complexes **1**–**3** according to eq 1, and semiquinone-bridged complexes **4** and **5** according to eq 2.

Compounds	**1** (M = Fe)	**2** (M = Co)	**3** (M = Ni)	**4** (M = Fe)	**5** (M = Co)
J (cm^{-1})	−0.847(2)	−2.595(5)	−5.94(1)	−144(1)	−252(2)
D_{ion} (cm^{-1})	−10.48(5)	−24.4(1)	−96.6(6)	7.78(5)	10.9(1)
$\|E_{ion}\|$ (cm^{-1})	2.91(6)	0.0(5)	8.3(1)	1.092(8)	3.23(3)
$g_{\parallel}$	2.220(2)	2.241(4)	2.163(5)	2.015(3)	2.475(1)[a]
$g_{\perp}$	2.233(3)	2.509(3)	2.960(5)	2.1799(7)	2.180(1)[a]
monomeric impurity (%)	–	5.4(1)	6.8(1)	–	–

[a] $g_{\parallel} = g_x$, while $g_{\perp} = g_y = g_z$

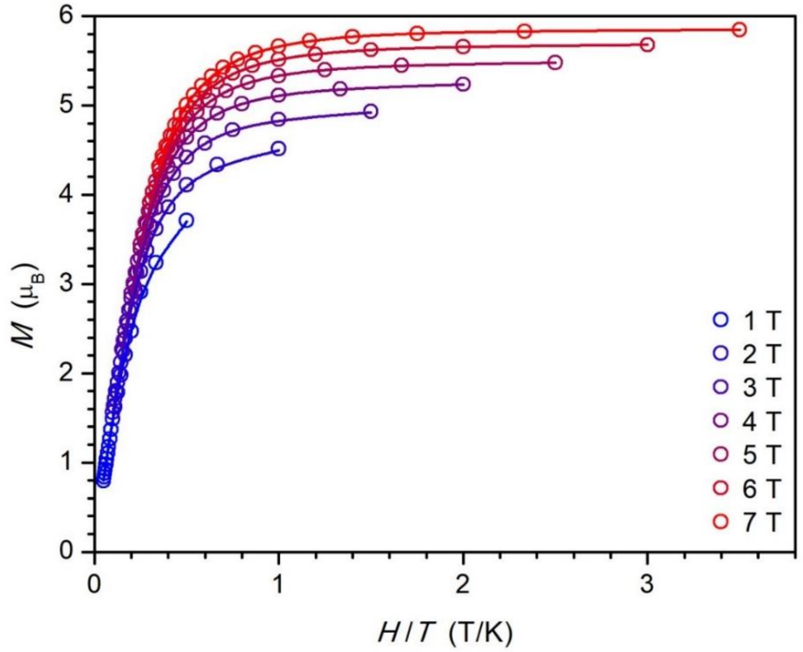

Figure S24. Reduced magnetization plot of **4** collected under fields of 1 to 7 T from 2 to 20 K. Solid lines are fits to the data according to eq 2 or S1.

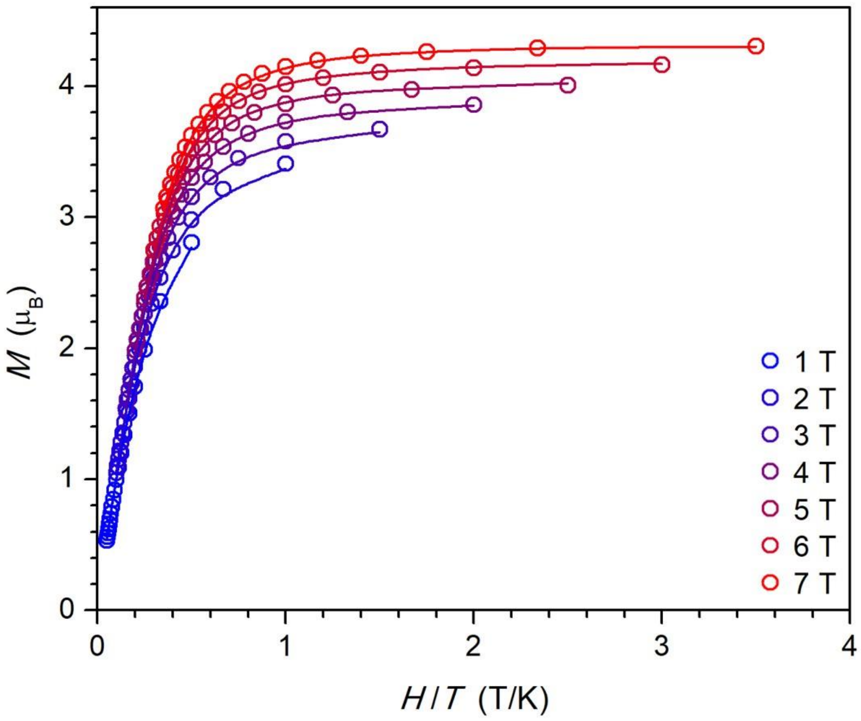

Figure S25. Reduced magnetization plot of **5** collected under fields of 1 to 7 T from 2 to 20 K. Solid lines are fits to the data according to eq 2 or S1.

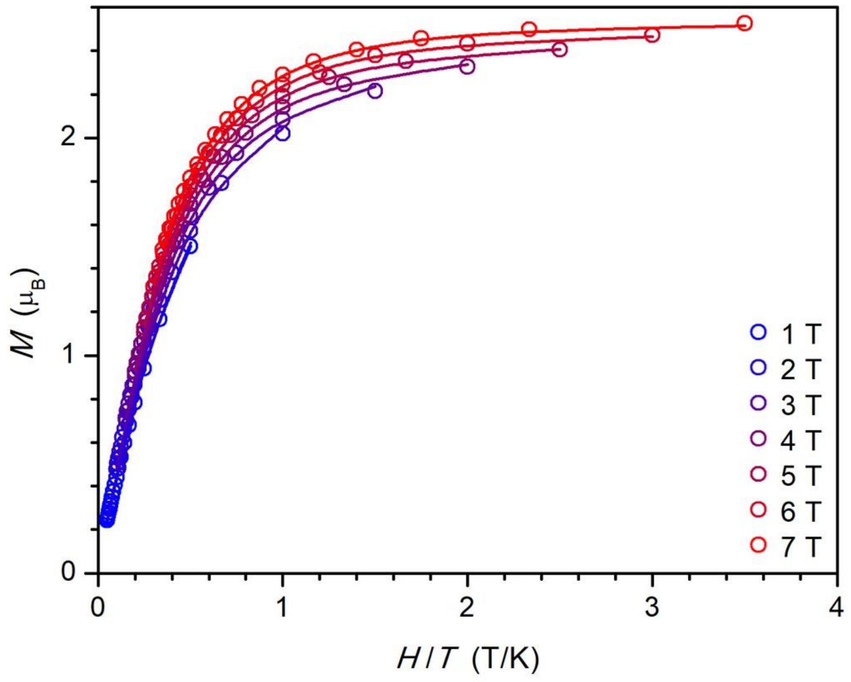

Figure S26. Reduced magnetization plot of **6** collected under fields of 1 to 7 T from 2 to 20 K. Solid lines are fits to the data according to eq S1.

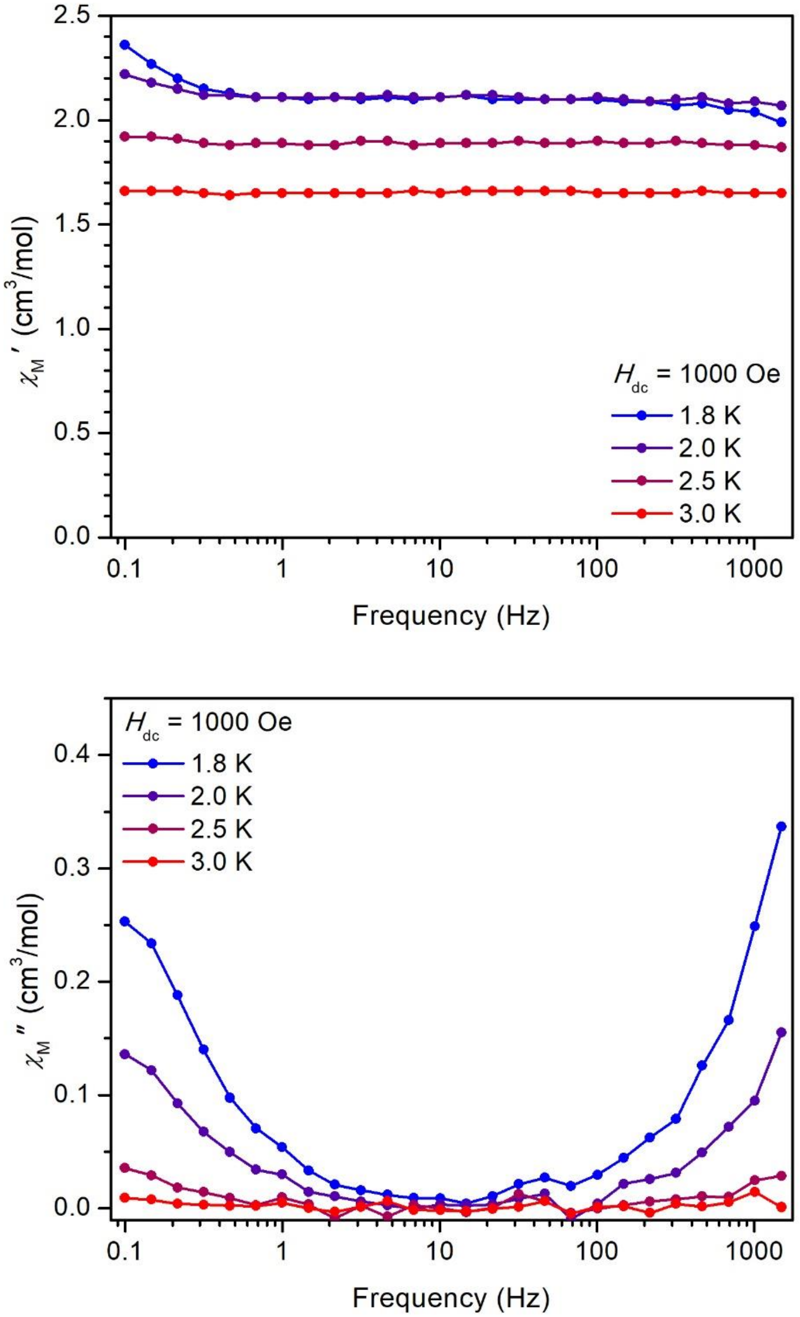

Figure S27. Variable-temperature, variable-frequency in-phase (top) and out-of-phase (bottom) components of the ac magnetic susceptibility data of **4** collected under a 1000 Oe applied magnetic field. Solid lines are guides for the eye.

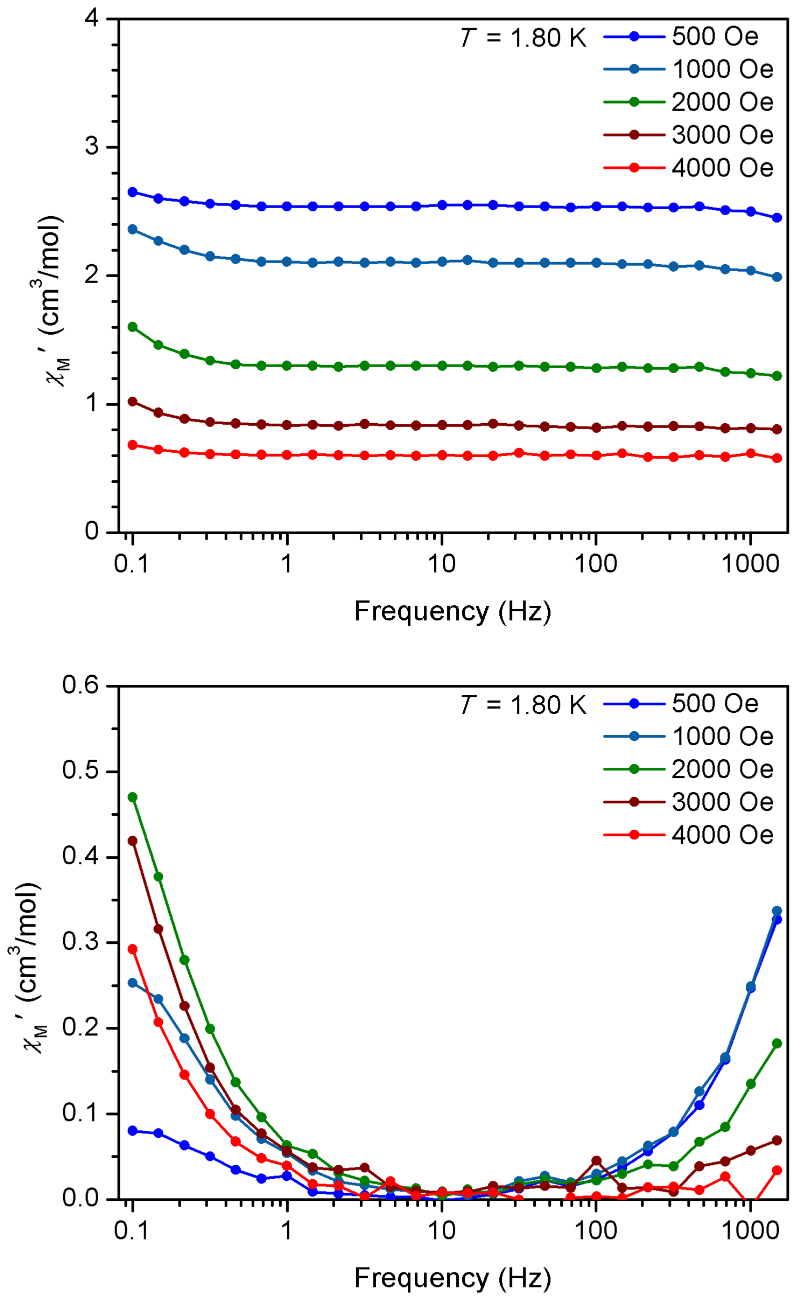

Figure S28. Variable-field, variable-frequency in-phase (top) and out-of-phase (bottom) components of the ac magnetic susceptibility data of **4** collected at 1.8 K. Solid lines represent fits to the data using a generalized Debye model for two relaxation processes.

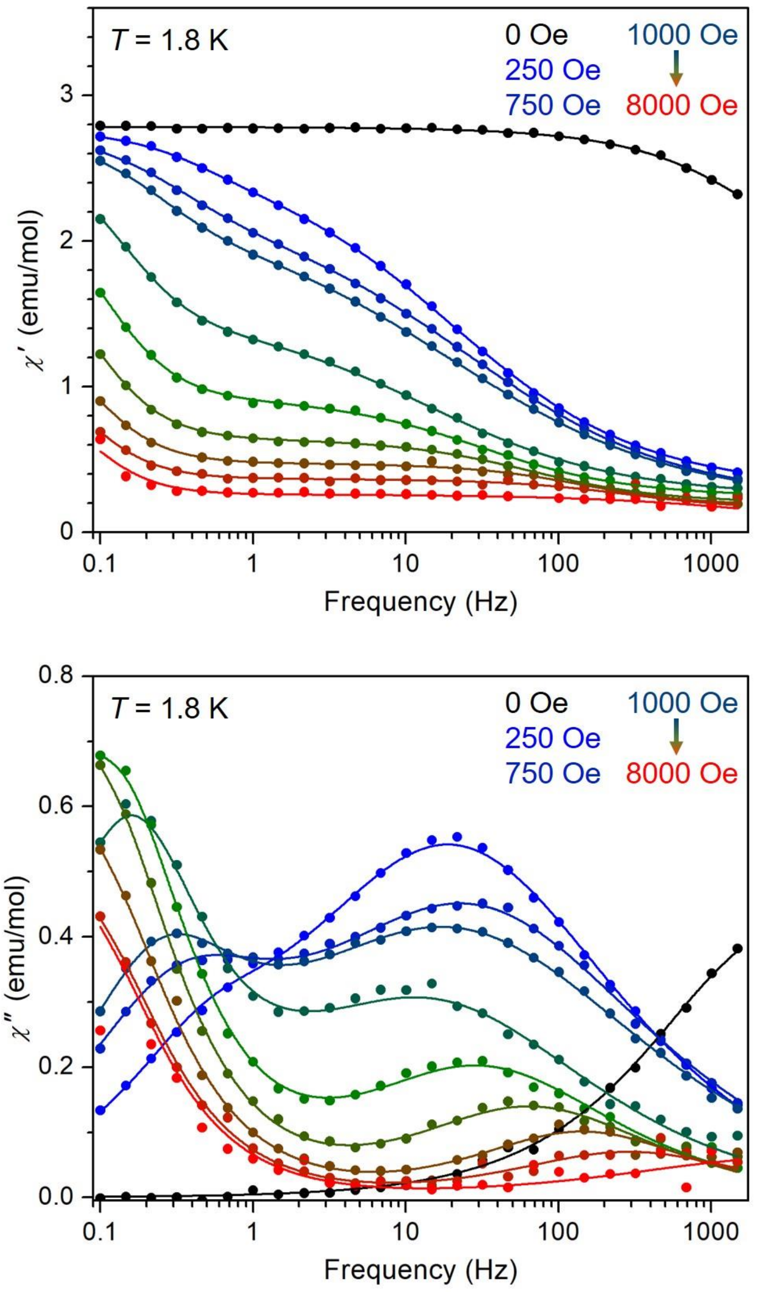

Figure S29. Variable-field, variable-frequency in-phase (top) and out-of-phase (bottom) components of the ac magnetic susceptibility data of **5** collected at 1.8 K. Solid lines represent fits to the data using a generalized Debye model for two relaxation processes.

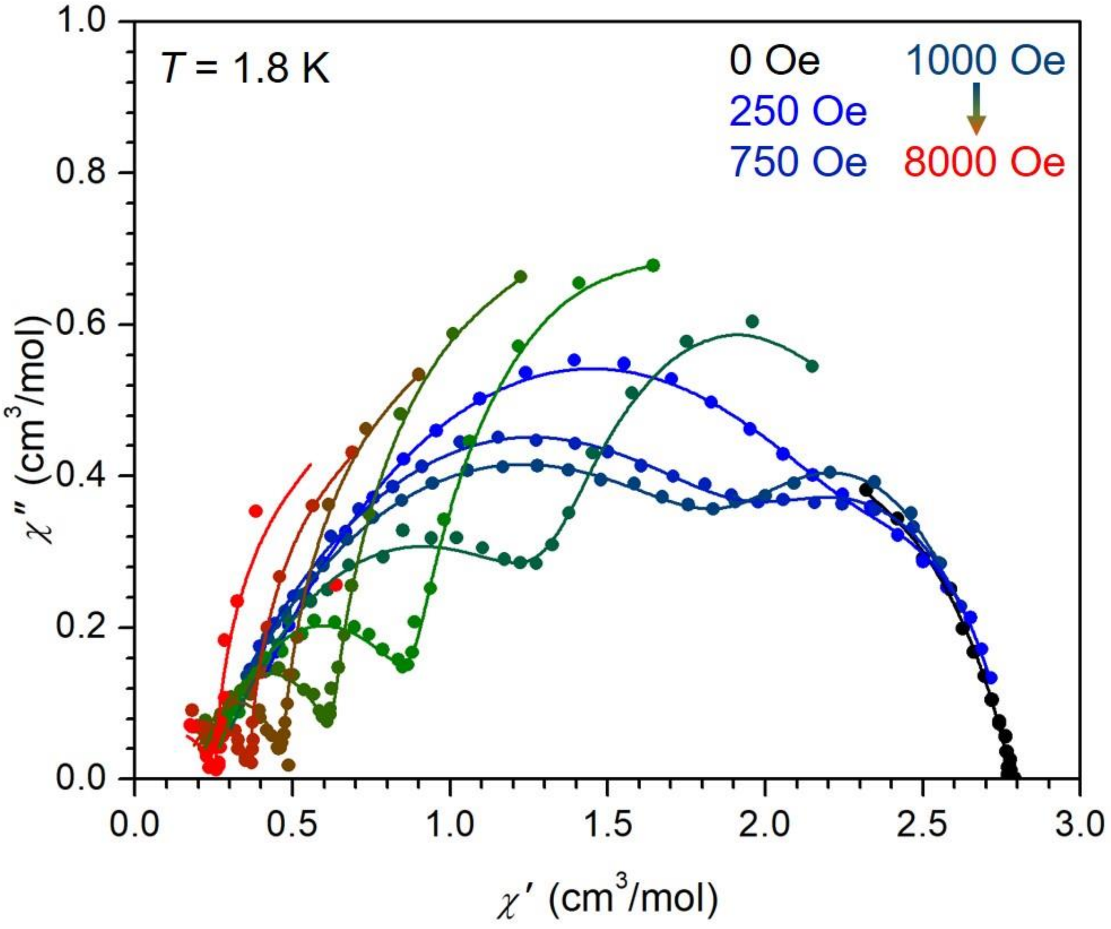

Figure S30. Variable-field Cole-Cole plots of **5** collected at 1.8 K. Solid lines represent fits to the data using a generalized Debye model for two relaxation processes.

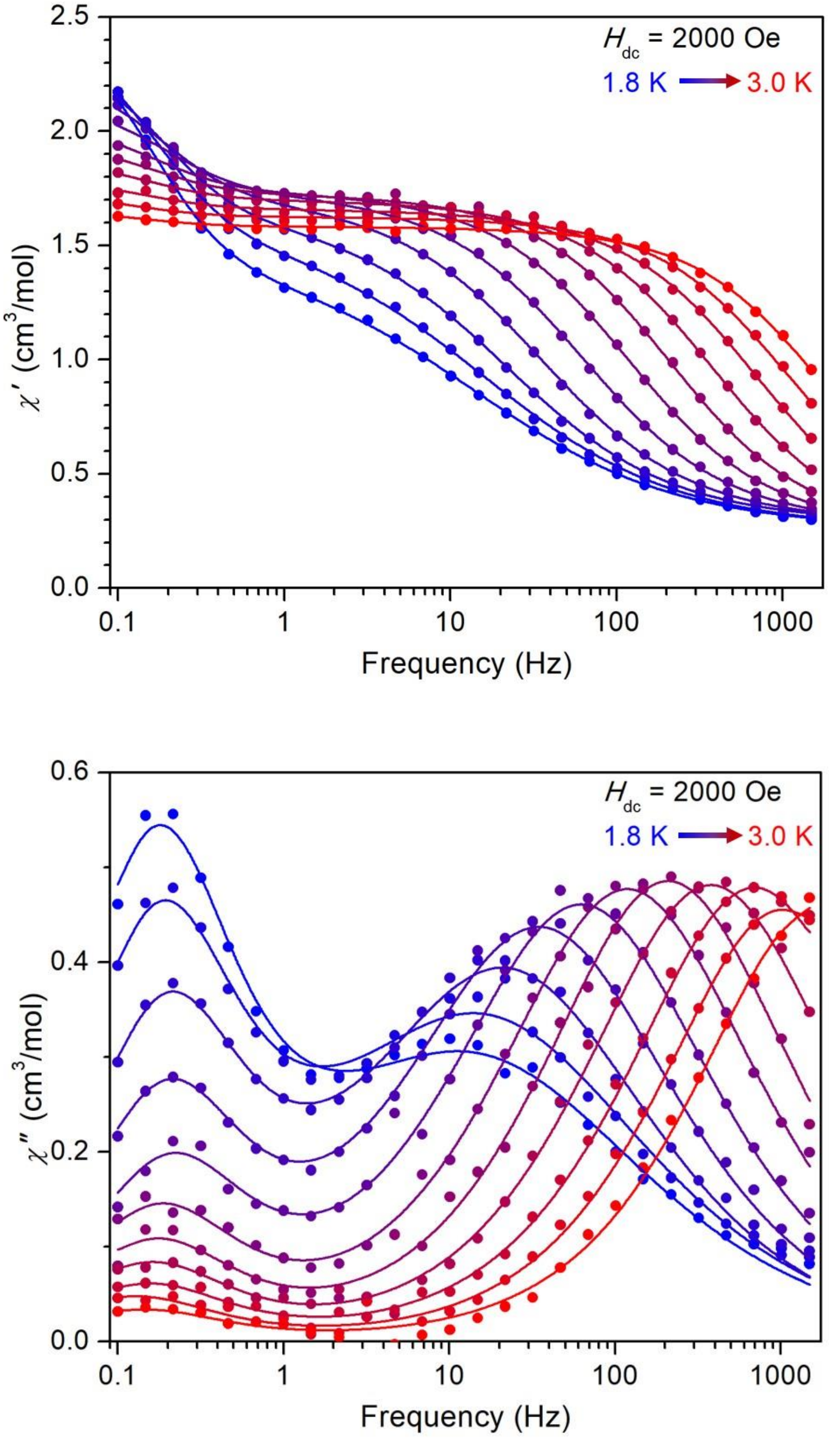

Figure S31. Variable-temperature, variable-frequency in-phase (top) and out-of-phase (bottom) components of the ac magnetic susceptibility data of **5** collected under a 2000 Oe applied magnetic field. Solid lines are fits to the data using a generalized Debye model for two relaxation processes.

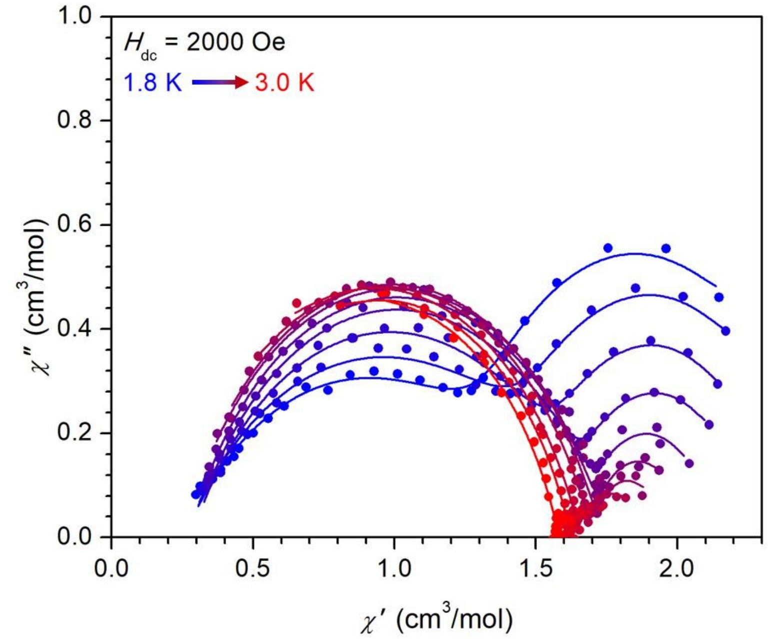

Figure S32. Variable-temperature Cole-Cole plots of **5** collected under a 2000 Oe applied field. Solid lines represent fits to the data using a generalized Debye model for two relaxation processes.

Table S8. Fitting parameters for the variable-temperature, variable-frequency ac susceptibility data of **5** under an applied field of 2000 Oe. The first process corresponds to the molecular spin relaxation, and the second process corresponds to intermolecular relaxation.

T (K)	χ_{T1} (cm^3/mol)	χ_{S1} (cm^3/mol)	α_1	τ_1 (ms)	χ_{T2} (cm^3/mol)	χ_{S2} (cm^3/mol)	α_2	τ_2 (s)	Residual $\times10^3$
1.80	1.45(2)	0.246(8)	0.42(1)	11.1(4)	0.98(1)	0	0	0.96(2)	5.72
1.90	1.59(2)	0.253(8)	0.41(1)	10.0(3)	0.81(1)	0	0	0.92(3)	6.49
2.00	1.653(7)	0.281(5)	0.341(6)	7.3(1)	0.650(8)	0	0	0.83(2)	2.85
2.12	1.717(6)	0.282(5)	0.302(6)	4.51(6)	0.497(8)	0	0	0.83(2)	3.42
2.26	1.739(7)	0.27(1)	0.282(9)	2.54(5)	0.36(1)	0	0	0.79(5)	8.89
2.38	1.740(4)	0.255(8)	0.268(6)	1.34(2)	0.274(8)	0	0	0.91(5)	3.71
2.50	1.727(5)	0.26(1)	0.251(9)	0.76(2)	0.21(1)	0	0	0.96(10)	8.35
2.62	1.699(3)	0.23(1)	0.257(6)	0.420(8)	0.163(9)	0	0	1.03(9)	3.60
2.75	1.659(4)	0.20(3)	0.25(1)	0.230(9)	0.12(1)	0	0	1.1(2)	6.53
2.88	1.625(3)	0.28(3)	0.24(1)	0.156(7)	0.09(1)	0	0	1.3(2)	5.17
3.00	1.582(3)	0.26(4)	0.23(1)	0.098(6)	0.07(1)	0	0	1.1(3)	4.26

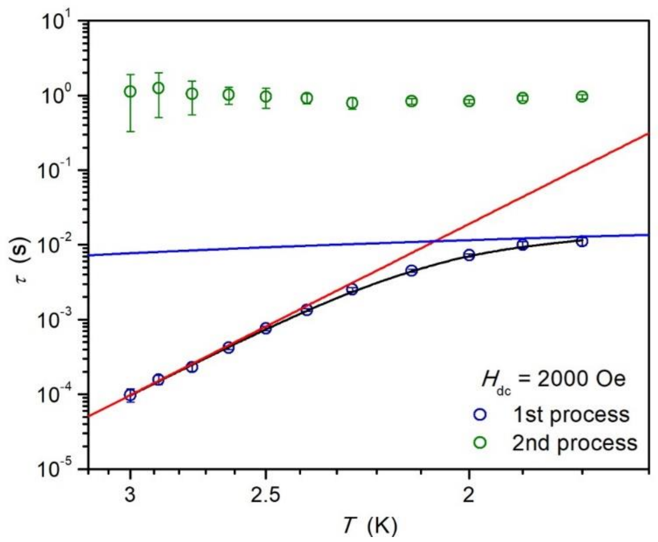

Figure S33. Arrhenius plots of the relaxation time τ (log scale) versus temperature (inverse scale) for the two relaxation processes observed for complex **5**. The first process (blue circles) exhibits temperature dependence that can be fit as a sum (black line) of contributions from Orbach (red line) and direct processes (blue line), as described in the main text. The second process (green circles) is almost temperature-independent and diminishes in magnitude with increasing temperature, characteristic of relaxation mediated by intermolecular interactions. The error bars represent three times the standard deviations of the data.

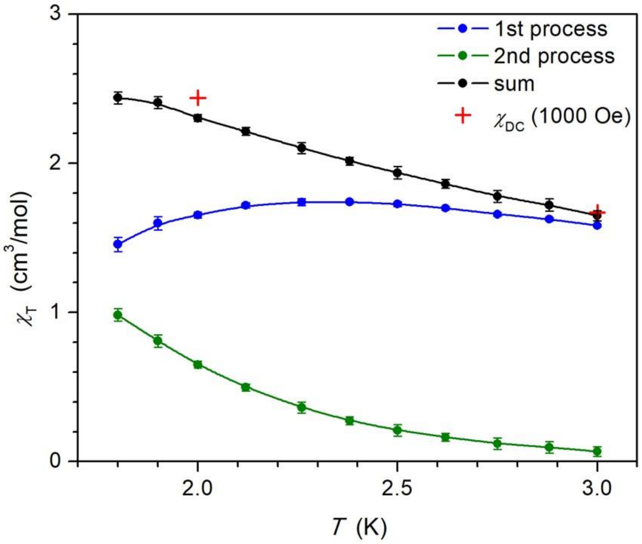

Figure S34. Isothermal susceptibility (χ_T) data versus T obtained from ac susceptibility measurements for molecular (blue circles) and intermolecular (green circles) relaxation processes occurring for **5** under a 2000 Oe field. The sum of the curves (black circles) yields data points that agree well with those obtained from the static molar susceptibility measurements under a 1000 Oe field (red crosses). Solid lines are guide to the eyes. The error bars represent three times the standard deviations of the data. The contribution from the second process diminishes with increasing temperature, as is typically observed for an intermolecular process.

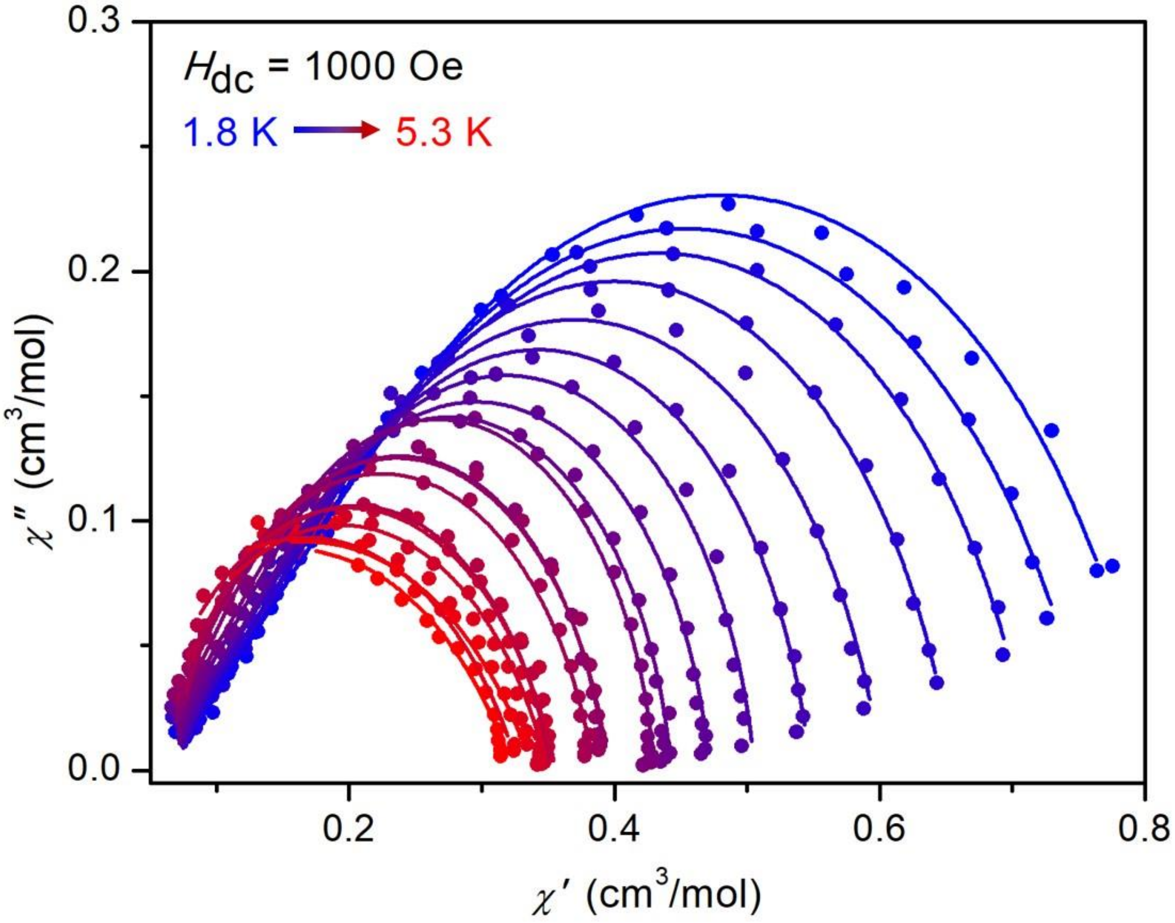

Figure S35. Variable-temperature Cole-Cole plots of **6** collected under a 1000 Oe applied field. Solid lines represent fits to the data using a generalized Debye model for two relaxation processes.

The ac data for the nickel(II) complex **6** features peak asymmetry suggesting the presence of non-uniform relaxation behavior, which might arise due to the two distinct $[(NiMe_6tren)_2(C_6H_4O_2)]^{3+}$ cations in the asymmetric unit of the crystal structure. However, the ratio of these two processes, as extracted from isothermal susceptibility (χ_T) data for the two species, does not coincide with the crystallographic occupancy of the two molecular cations. The second process might also arise as a result of disorder present in the second molecule in the structure. We note that the sum of χ_T for the two processes does not coincide with the static magnetic susceptibility data (Figure S38), which suggests that there is additional slow relaxation unobserved on the timescale of the ac measurement.

Table S9. Fitting parameters for ac susceptibility data collected for **6** under an applied field of 1000 Oe. Processes 1 is the major process observed.

T (K)	χ_{T1} (cm^3/mol)	χ_{S1} (cm^3/mol)	α_1	τ_1 (ms)	χ_{T2} (cm^3/mol)	χ_{S2} (cm^3/mol)	α_2	τ_2 (ms)	Residual ×10^3
1.80	0.64(17)	0.058(13)	0.19(6)	158(11)	0.17(19)	0	0.5(2)	9(24)	2.26
2.00	0.62(7)	0.069(6)	0.19(2)	124(6)	0.14(7)	0	0.5(1)	7(8)	0.62
2.16	0.56(7)	0.068(5)	0.17(3)	109(5)	0.15(7)	0	0.4(1)	7(7)	0.69
2.34	0.52(6)	0.069(4)	0.14(2)	90(4)	0.14(6)	0	0.39(9)	7(5)	0.59
2.50	0.48(6)	0.064(6)	0.13(3)	69(4)	0.13(6)	0	0.4(1)	4(4)	0.79
2.75	0.43(5)	0.070(4)	0.09(3)	55(3)	0.12(5)	0	0.3(1)	5(3)	0.80
3.00	0.38(11)	0.067(7)	0.07(7)	38(4)	0.13(12)	0	0.3(2)	4(7)	2.03
3.25	0.38(4)	0.064(5)	0.09(2)	25(1)	0.09(4)	0	0.3(1)	2(1)	0.48
3.50	0.39(2)	0.068(3)	0.09(1)	15.5(7)	0.06(2)	0	0.2(1)	1.1(4)	0.46
3.75	0.38(2)	0.067(4)	0.08(2)	8.7(4)	0.05(2)	0	0.1(1)	0.6(2)	0.63
4.00	0.397(2)	0.069(2)	0.17(1)	4.05(9)	–	–	–	–	0.89
4.16	0.398(2)	0.065(3)	0.17(1)	2.55(6)	–	–	–	–	0.76
4.33	0.384(1)	0.064(2)	0.178(8)	1.63(3)	–	–	–	–	0.35
4.50	0.353(1)	0.057(2)	0.201(8)	0.98(2)	–	–	–	–	0.32
4.67	0.356(1)	0.044(4)	0.24(1)	0.57(2)	–	–	–	–	0.51
4.84	0.350(2)	0.039(7)	0.28(1)	0.36(2)	–	–	–	–	0.59
5.00	0.328(1)	0	0.333(9)	0.189(4)	–	–	–	–	0.48
5.17	0.339(3)	0	0.36(1)	0.130(4)	–	–	–	–	0.49
5.33	0.324(2)	0	0.36(1)	0.088(3)	–	–	–	–	0.30

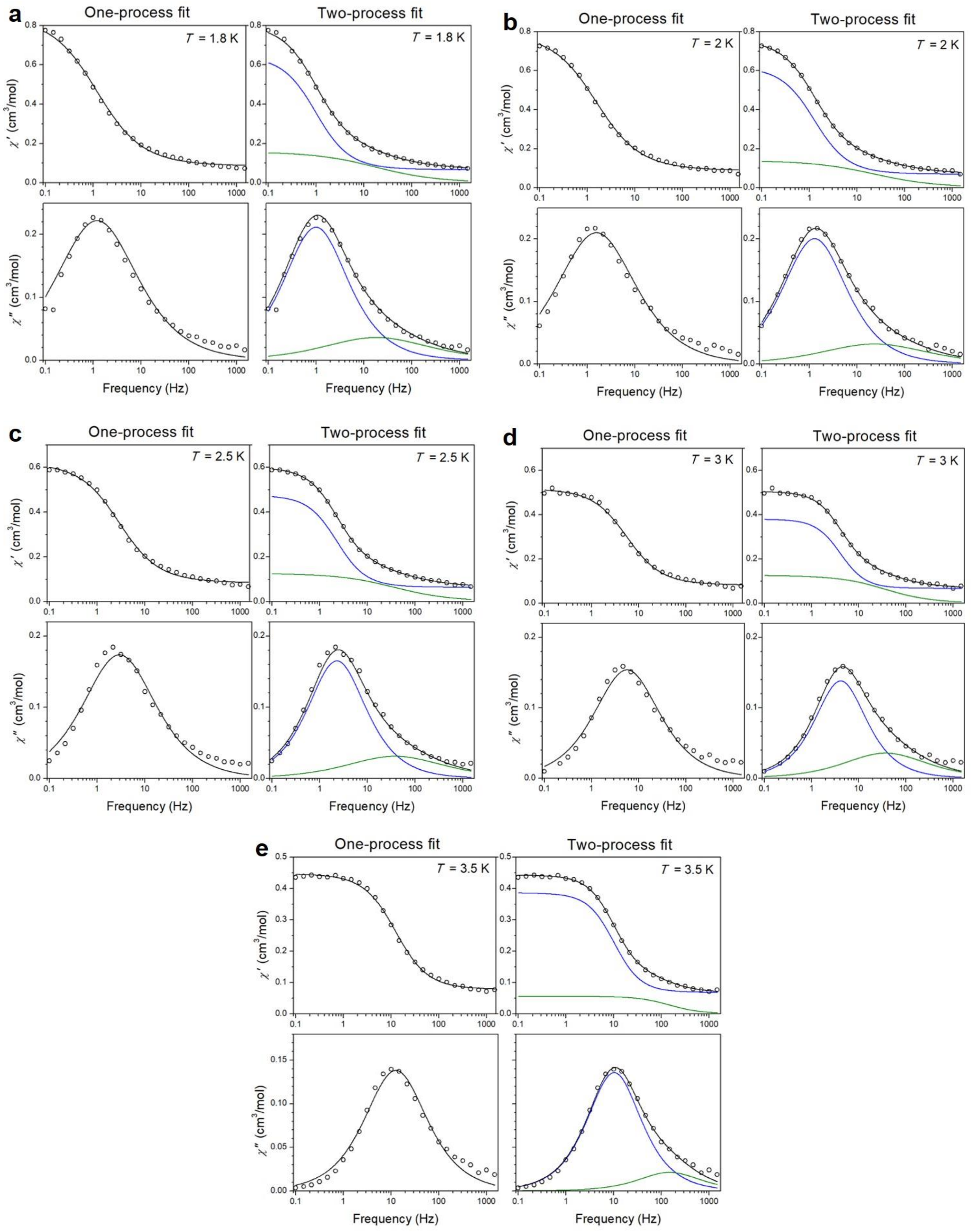

Figure S36. Comparison of selected ac susceptibility data of **6** under 1000 Oe applied field fit to the generalized Debye model with one (left) versus two (right) relaxation processes. Blue, green, and black lines represent fits to the first and second process, and the overall fits, respectively. Selected data and fit are shown for T = 1.8 K (**a**), 2 K (**b**), 2.5 K (**c**), 3 K (**d**), and 3.5 K (**e**).

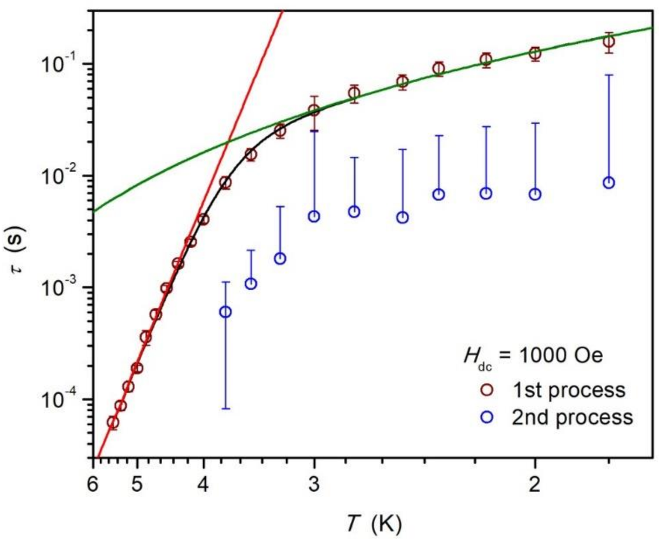

Figure S37. Arrhenius plots of relaxation time τ (log scale) versus T (inverse scale) for the two ac relaxation processes observed for **6**. The first process (dark red circles) shows temperature-dependent behavior characteristic of a single-molecule magnet. Black line represents the total fit to the data according to eq 3 in the main text. Red and green lines represent Orbach and Raman relaxations, respectively. The second process (blue circles) exhibits similar temperature dependence. The error bars represent three times the standard deviations of the data.

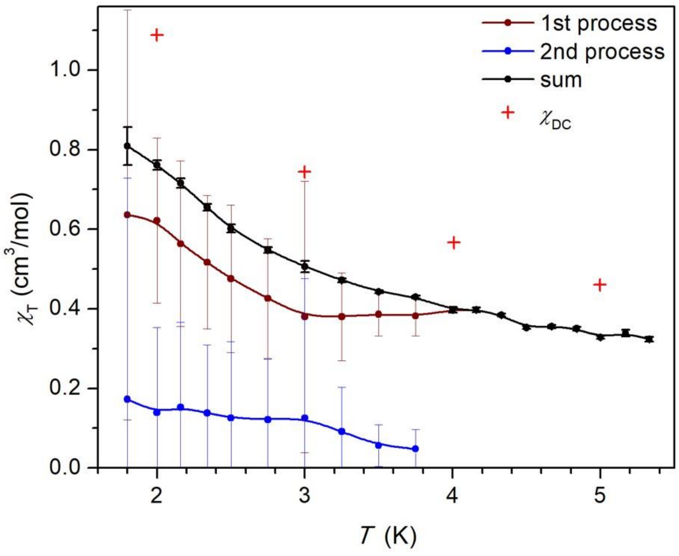

Figure S38. Isothermal susceptibility (χ_T) data as measured by ac susceptometry for the first and second relaxation processes in **6** under a 1000 Oe field, plotted as dark red and blue circles, respectively. The error bars represent three times the standard deviations of the data. The sum of the χ_T data (black circles) does not coincide with the static molar magnetic susceptibility (red crosses), indicating that there is an additional slow magnetic relaxation process that is observed on the measured ac timescale. Solid lines are guide to the eyes.

References

(1) Ciampolini, M.; Nardi, N. Trigonal Bipyramidal Complexes of Bivalent Manganese Iron, and Zinc with Tris(2-dimethylaminoethyl)amine. *Inorg. Chem.* **1966**, *5* (7), 1150–1154.

(2) Ciampolini, M.; Nardi, N. Five-Coordinated High- Spin Complexes of Bivalent Cobalt, Nickel, and Copper with Tris(2-dimethylaminoethyl)amine. *Inorg. Chem.* **1966**, *5* (1), 41–44.

(3) Adams, J. J.; Arulsamy, N.; Sullivan, B. P.; Roddick, D. M.; Neuberger, A.; Schmehl, R. H. Homoleptic Tris-Diphosphine Re(I) and Re(II) Complexes and Re(II) Photophysics and Photochemistry. *Inorg. Chem.* **2015**, *54*, 11136–11149.

(4) SAINT and APEX 2 Software for CCD Diffractometers, Bruker AXS Inc., Madison, WI. 2014.

(5) Sheldrick, G. M. SADABS, Version 2.03, Bruker Analytical X-Ray Systems, Inc., Madison, WI. Bruker Analytical X-Ray Systems, Inc.: Madison, WI 2000.

(6) Sheldrick, G. M. SHELXT – Integrated Space-Group and Crystal-Structure Determination. *Acta Crystallogr. A* **2015**, *71*, 3–8.

(7) Sheldrick, G. M. A Short History of SHELX. *Acta Crystallogr. A* **2008**, *64*, 112–122.

(8) Dolomanov, O. V.; Bourhis, L. J.; Gildea, R. J.; Howard, J. A. K.; Puschmann, H. OLEX2 : A Complete Structure Solution, Refinement and Analysis Program. *J. Appl. Crystallogr.* **2009**, *42*, 339–341.

(9) Comba, P.; Hausberg, S.; Martin, B. Calculation of Exchange Coupling Constants of Transition Metal Complexes with DFT. *J. Phys. Chem. A* **2009**, *113* (24), 6751–6755.

(10) Ruiz, E.; Cano, J.; Alvarez, S.; Alemany, P. Broken Symmetry Approach to Calculation of Exchange Coupling Constants for Homobinuclear and Heterobinuclear Transition Metal Complexes. *J. Comput. Chem.* **1999**, *20* (13), 1391–1400.

(11) Hazra, S.; Sasmal, S.; Fleck, M.; Grandjean, F.; Sougrati, M. T.; Ghosh, M.; Harris, T. D.; Bonville, P.; Long, G. J.; Mohanta, S. Slow Magnetic Relaxation and Electron Delocalization in an S = 9⁄2 Iron(II⁄III) Complex with Two Crystallographically Inequivalent Iron Sites. *J. Chem. Phys.* **2011**, *134*, 174507.

(12) Zadrozny, J. M.; Xiao, D. J.; Long, J. R.; Atanasov, M.; Neese, F.; Grandjean, F.; Long, G. J. Mössbauer Spectroscopy as a Probe of Magnetization Dynamics in the Linear Iron(I) and Iron(II) Complexes $[Fe(C(SiMe_3)_3)_2]^{1-/0}$. *Inorg. Chem.* **2013**, *52*, 13123–13131.

(13) Gütlich, P.; Bill, E.; Trautwein, A. X. *Mössbauer Spectroscopy and Transition Metal Chemistry*; Springer Berlin Heidelberg: Berlin, Heidelberg, 2011.

(14) Bain, G. A.; Berry, J. F. Diamagnetic Corrections and Pascal's Constants. *J. Chem. Educ.* **2008**, *85*, 532–536.

(15) Chilton, N. F.; Anderson, R. P.; Turner, L. D.; Soncini, A.; Murray, K. S. PHI: A Powerful New Program for the Analysis of Anisotropic Monomeric and Exchange-Coupled Polynuclear d - and f -Block Complexes. *J. Comput. Chem.* **2013**, *34*, 1164–1175.

(16) Gatteschi, D.; Sessoli, R.; Villain, J. *Molecular Nanomagnets*; Oxford Univ. Press, 2006.

(17) SolverAid macro is available from the MacroBundle developed by Prof. Robert de Levie. www.bowdoin.edu/~rdelevie/excellaneous
